# Developing a measure of polypharmacy appropriateness in primary care: systematic review and expert consensus study

**DOI:** 10.1186/s12916-018-1078-7

**Published:** 2018-06-13

**Authors:** Jenni Burt, Natasha Elmore, Stephen M. Campbell, Sarah Rodgers, Anthony J. Avery, Rupert A. Payne

**Affiliations:** 1 THIS Institute (The Healthcare Improvement Studies Institute), University of Cambridge, Cambridge Biomedical Campus, Clifford Allbutt Building, Cambridge, CB2 0AH UK; 2 NIHR Greater Manchester Patient Safety Translational Research Centre, Division of Population Health, HSR & Primary Care, School of Health Sciences, Faculty of Biology, Medicine and Health, University of Manchester, Manchester, UK; 3 Division of Primary Care, University of Nottingham, Room 1312, Tower Building, University Park, Nottingham, NG7 2RD UK; 4 Division of Primary Care, School of Medicine, University of Nottingham, Dean’s Office, B Floor, Medical School, Queens Medical Centre, Nottingham, NG7 2UH UK; 5 Centre for Academic Primary Care, Population Health Sciences, Bristol Medical School, University of Bristol, Canynge Hall, 39 Whatley Road, Bristol, BS8 2PS UK

**Keywords:** Inappropriate prescribing, polypharmacy, medication errors, multimorbidity, primary care, consensus methods, systematic review

## Abstract

**Background:**

Polypharmacy is an increasing challenge for primary care. Although sometimes clinically justified, polypharmacy can be inappropriate, leading to undesirable outcomes. Optimising care for polypharmacy necessitates effective targeting and monitoring of interventions. This requires a valid, reliable measure of polypharmacy, relevant for all patients, that considers clinical appropriateness and generic prescribing issues applicable across all medications. Whilst there are several existing measures of potentially inappropriate prescribing, these are not specifically designed with polypharmacy in mind, can require extensive clinical input to complete, and often cover a limited number of drugs. The aim of this study was to identify what experts consider to be the key elements of a measure of prescribing appropriateness in the context of polypharmacy.

**Methods:**

Firstly, we conducted a systematic review to identify generic (not drug specific) prescribing indicators relevant to polypharmacy appropriateness. Indicators were subject to content analysis to enable categorisation. Secondly, we convened a panel of 10 clinical experts to review the identified indicators and assess their relative clinical importance. For each indicator category, a brief evidence summary was developed, based on relevant clinical and indicator literature, clinical guidance, and opinions obtained from a separate patient discussion panel. A two-stage RAND/UCLA Appropriateness Method was used to reach consensus amongst the panel on a core set of indicators of polypharmacy appropriateness.

**Results:**

We identified 20,879 papers for title/abstract screening, obtaining 273 full papers. We extracted 189 generic indicators, and presented 160 to the panel grouped into 18 classifications (e.g. adherence, dosage, clinical efficacy). After two stages, during which the panel introduced 18 additional indicators, there was consensus that 134 indicators were of clinical importance. Following the application of decision rules and further panel consultation, 12 indicators were placed into the final selection. Panel members particularly valued indicators concerned with adverse drug reactions, contraindications, drug-drug interactions, and the conduct of medication reviews.

**Conclusions:**

We have identified a set of 12 indicators of clinical importance considered relevant to polypharmacy appropriateness. Use of these indicators in clinical practice and informatics systems is dependent on their operationalisation and their utility (e.g. risk stratification, targeting and monitoring polypharmacy interventions) requires subsequent evaluation.

**Trial registration:**

Registration number: PROSPERO (CRD42016049176).

**Electronic supplementary material:**

The online version of this article (10.1186/s12916-018-1078-7) contains supplementary material, which is available to authorized users.

## Background

The use of multiple medications in a single individual (polypharmacy) is a global phenomenon, creating new challenges for many health services [1], driven by increasing levels of multimorbidity [2] and a culture of single-condition guideline-based prescribing [3].

Polypharmacy is associated with several undesirable consequences [4–8]. However, we have previously demonstrated that the association between polypharmacy and adverse outcomes is attenuated in the most multimorbid individuals [9]. This suggests that overly simplistic analyses of polypharmacy, relating simple medication counts to adverse outcomes, may be misleading [9, 10]. Polypharmacy is typically measured using arbitrary numeric thresholds, but these fail to capture medication appropriateness; therefore, more sophisticated approaches accounting for clinical context are required.

A number of prescribing indicators that assess medication appropriateness are considered to have face validity [11], and may have value in improving quality and reducing adverse outcomes [12]. However, such indicators generally do not account for multiple drug use and do not measure polypharmacy per se. In addition, explicit ‘drug specific’ indicators (e.g. Beers criteria [13]) do not apply to all patients, and implicit measures (e.g. the Medication Appropriateness Index [14]) require time consuming input from an experienced clinician.

There is therefore a need to develop a valid and reliable means of measuring polypharmacy that takes account of clinical appropriateness. Further, a framing of polypharmacy appropriateness (rather than appropriate or inappropriate polypharmacy) acknowledges a spectrum of prescribing within the context of polypharmacy, including the need to support individualised prescribing approaches where potentially risky (or ‘inappropriate’) combinations may be fitting for a particular patient. To be usable in clinical practice, this measure should ideally focus on generic prescribing issues (to ensure relevance to all patients, and avoiding simply focusing on a finite number of medications, i.e. implicit indicators), whilst still permitting automation as part of a computerised clinical system. We anticipate that this measure of polypharmacy appropriateness would enable more effective targeting and evaluation of medication optimisation interventions. Used as a first step in identifying which patients may be at risk of inappropriate prescribing, such a measure could facilitate targeted conversations with patients to subsequently ascertain their views on the appropriateness of their current medication regimen.

## Methods

The aim of this study was to identify the key elements of a measure of prescribing appropriateness in the context of polypharmacy. We conducted a systematic review and RAND/UCLA Appropriateness Method consensus study, as outlined below. Whilst our aim was to develop a measure of polypharmacy appropriateness, the orientation of most measures to date has been one of inappropriate prescribing, and we therefore adopt that terminology when referencing existing approaches.

### Systematic review of indicators of polypharmacy appropriateness

To locate and derive implicit indicators of polypharmacy appropriateness for consideration by the expert panel, an initial systematic review was undertaken*.* The protocol was prospectively registered with PROSPERO (registration number CRD42016049176) [15, 16] and the manuscript was written in accordance with the PRISMA statement [17].

#### Search strategy

We searched Embase, MEDLINE (Ovid), PsycINFO, CINAHL, Health Management Information Consortium, Cochrane Library, Web of Science, the Trip and NHS Evidence databases, from 1992 (the year the Medication Appropriateness Index was first published [14]) until October 10, 2016. Search terms were developed in collaboration with an experienced medical librarian. We used exploded MeSH terms (e.g. inappropriate prescribing) and combinations of relevant keywords and their variants (e.g. medication, drug therapy, drug utilisation, drug utilisation review, prescribing). We adapted our initial MEDLINE search strategy (Box 1) to run in the additional databases, and to perform a review of reviews to identify any previous reviews in this field (Box 2). Further relevant publications were identified by a manual search of references and citations of included papers.

#### Inclusion criteria

Articles were eligible for inclusion if they reported the use of a specific tool to assess polypharmacy or inappropriate prescribing, including both implicit and explicit indicators. We defined ‘implicit’ (or ‘generic’) indicators as judgement-based indicators that facilitate the consideration of the whole patient, rather than having a narrow focus on specific drugs or diseases [11]. We defined ‘explicit’ indicators as criterion-based indicators that assess specific drug-based or disease-based prescribing against specific standards derived from guidelines or expert opinion [11].

The decision to include both implicit and explicit indicators at full-text screening, rather than including only articles stating the focus was on implicit indicators, was based on our observation that implicit indicators were not always clearly identified as such. For example, a single implicit indicator may be embedded within instruments labelled as explicit indicators, as is the case with the guidelines developed by Basger et al. [18, 19] in the Australian context. We excluded papers describing non-instrument based medication reviews, educational interventions, validation studies of previously published tools, general guidelines and recommendations relating to assessing inappropriate prescribing, and articles not published in English.

#### Screening

Publications were assessed for eligibility by title and abstract screening. An initial random sample of 100 titles/abstracts was triple screened to refine the application of inclusion and exclusion criteria. A single reviewer (NE) subsequently screened all titles/abstracts, with a 10% random sample screened by two additional reviewers (JB, RP). Any disagreements were resolved by discussion between the three reviewers. Articles included for full text screening were retrieved and reviewed against the inclusion criteria by two reviewers (NE and JB) independently, with discrepancies resolved by discussion with RP.

#### Extraction of indicators

All full-text papers meeting the inclusion criteria were reviewed to find implicit indicators of polypharmacy or inappropriate prescribing. Indicators were extracted and subjected to qualitative content analysis to identify categories into which they could be grouped [20]. Two authors (JB and RP) independently reviewed all indicators and generated a coding framework, which was refined through consensus discussions to produce a final set of indicator categories. Indicators that, on further scrutiny, were deemed not to be truly implicit were excluded at this stage. All indicators, within their categories, were subsequently taken forward for expert review in a consensus panel.

#### Quality assessment of indicators

Where stated, the development method for the identified indicators was extracted, and its strengths and limitations were assessed with reference to the Joanna Briggs Institute critical appraisal checklist for text and opinion [21].

### Expert consensus process

The two-stage RAND/UCLA Appropriateness Method was employed to reach consensus on indicators of prescribing considered by experts to be key to a measure of polypharmacy appropriateness. The RAND/UCLA Appropriateness Method is a well-established approach for systematically generating expert consensus by combining scientific evidence with expert opinion, and subsequently aggregating individual opinions into a single perspective [22].

#### Patient views

We convened a panel of five patient representatives from a local ‘patient and public involvement in research’ group to gather their views on which aspects of polypharmacy and prescribing identified by the systematic review were of particular relevance and concern to them. The panel meeting was facilitated by a general practitioner (RP), with support from a specialist Patient and Public Involvement coordinator responsible for facilitating patient involvement in research [23]. The categories of polypharmacy or inappropriate prescribing into which the identified indicators had been grouped were presented to the panel, and discussion encouraged around the particular importance or relevance of this area to the patient experience. Patient representatives were asked, in particular, to reflect on the relevance and importance of each prescribing indicator category, whether doing something about it would make a difference, the potential challenges of measuring it, and whether they would prioritise one or more categories. Field notes were taken during the conduct of the panel meeting and key points across each category were summarised. A synopsis of the patient views in relation to each category was presented in narrative form in the evidence summaries supplied to the expert panel (see below).

#### Evidence summaries

For each indicator category, the research team developed a corresponding brief evidence summary. These summaries drew upon clinical and academic literature, and addressed the importance and relevance of the issue in relation to polypharmacy, the potential consequences for patients, and how the indicators had been developed and evaluated. As outlined above, patient views were also incorporated based on the earlier patient panel discussion. Evidence summaries were then presented to panel members to support discussions as part of the RAND/UCLA Appropriateness Method consensus exercise; these included a brief summary of the strengths and weaknesses of the approach to deriving each included indicator, as derived from the quality assessment of indicators.

#### Expert panel members

We undertook a snowball approach to recruit expert panel members, aiming to include a range of professions and levels of experience. We particularly focused on individuals immersed in the general prescribing process and those who regularly dealt with polypharmacy; we therefore targeted general practice, pharmacy, pharmacology, and geriatric medicine. We therefore approached relevant professional organisations, including the Royal College of General Practitioners, Royal Pharmaceutical Society, British Pharmacological Society, and additional known contacts of the research team. Our final ten-member panel consisted of four general practitioners, two pharmacists, three geriatricians, and one clinical pharmacologist; RAND/UCLA approaches typically include anything from 7 to 15 members in a panel [22]. Panel members were not involved in the systematic review itself.

#### First stage assessment

In stage one, we asked panel members to participate in an online survey to evaluate the implicit indicators of inappropriate prescribing or polypharmacy identified through the systematic review. Panel members were sent a personalised link to complete the first stage of ratings using the survey software, Qualtrics (https://www.qualtrics.com). Indicators were presented to panel members in their category groupings. Panel members were asked to review the indicators within each category and score each indicator in turn in relation to (1) its clinical importance with regards to polypharmacy and (2) its clarity, using a rating scale numbered 1 to 9, where a score of 1 meant the indicator was extremely unimportant (or unclear) in evaluating polypharmacy appropriateness, and a score of 9 meant the indicator was extremely important (and clear). Consensus classifications for each indicator were established using a series of rules, outlined in Table 1.

**Table 1:** Rules used to determine consensus classification for panel ratings

Median panel score	Criteria for:		
	Disagreement	Equivocal	Agreement
		> 20% of individual scores equal to:	20% of individual scores equal to:
1		4–9	4–9
2		5–9	5–9
3		6–9	6–9
4	≥ 33% of individual scores equal to 1–3 AND ≥ 33% of individual scores equal to 7–9	1, 7–9	1, 7–9
5		1, 2, 8, 9	1, 2, 8, 9
6		1–3, 9	1–3, 9
7		1–4	1–4
8		1–5	1–5
9		1–6	1–6

#### Second stage assessment

In stage two, we convened a face-to-face meeting of all panel members to further review and discuss indicators and their scores. The meeting was facilitated by an experienced RAND/UCLA Appropriateness Method facilitator (SC) with input from a general practitioner with expertise in polypharmacy (RP). Anonymised frequency distributions and median scores for the clinical importance rating of every stage one indicator were provided to each panel member at the start of the meeting, alongside the scores they had themselves assigned to every indicator. Categories of indicator were discussed, in turn, with panel members given the opportunity to suggest changes to the wording of the indicators; the wording of all new variations of indicators needed to be agreed by all panellists. At the end of discussions around each category, panel members were asked to rate each indicator again, including new variations, in relation to their clinical importance to polypharmacy.

#### Application of final decision rules

As indicators considered by the panel were presented in categorical groupings, many indicators, whilst drawn from different sources, were closely related. To ensure our final indicator set was a non-duplicative measure of polypharmacy appropriateness, we took the following steps. First, in order to decide which of the highest scoring indicators within each category to take forward to the final set, two members of the research team (JB and RP) independently coded indicators within each category grouping to identify sub-category groupings, which were further refined through discussion with two other team members (AA and SR). The highest scoring indicator from each sub-category grouping was subsequently taken forward; all such indicators scored a minimum of 7. Where more than one indicator in each sub-category had the same highest score, the decision as to which indicator to select was made within the research team through discussion and consideration of the likely feasibility of the operationalisation of that indicator in primary care. We then applied the following decision rules to the remaining indicators:

1. Able to be applied at the level of individual drugs
2. Not overlapping with or duplicating another indicator

The final suggested list was circulated to the panel members for any last comments and review.

## Results

### Systematic review

**Fig. 1:**
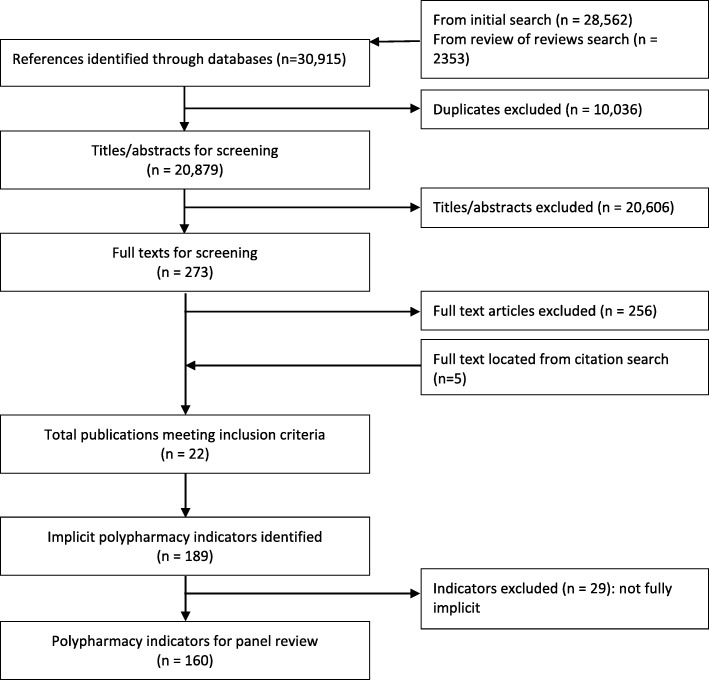
PRISMA Diagram showing review process

Our review identified 20,879 papers for title/abstract screening, from which 273 full-text papers were identified for review (Fig. 1). Double screening of a 10% random sample of all citations had a 97% consensus rate, with both reviewers making the same decision. Following full text screening, we selected 17 papers that included either all implicit or a mixture of implicit and explicit indicators. A further five papers were identified through forward and backward citation searching of the 17 papers. From the resulting 22 papers (Appendix 1), we identified 189 potential implicit indicators of polypharmacy or inappropriate prescribing. On further scrutiny, 29 indicators did not meet our inclusion criteria. The remaining 160 indicators (Additional file 1) were placed into 18 categories derived through content analysis (Box 3). The wording of each indicator remained unchanged from its original source and thus, at this stage, categories could contain similar indicators varying only slightly in their wording or grammatical construction.

### Patient panel

Although patient representatives discussed all of the prescribing indicator categories, they prioritised adherence, drug–drug interactions and clinical efficacy as fundamental to polypharmacy appropriateness. They raised concerns about how feasible it was to evaluate adherence, but felt it was a central issue for patients managing complex medication regimens. The avoidance of adverse drug–.drug interactions was also seen as vital; the patient panel stated that they looked to clinical expertise to evaluate and avoid these risks for patients. Finally, discussions on the role of clinical efficacy focused on how specific this was to the individual, and patient representatives voiced concerns raised about how clinicians could evaluate and optimise the overall efficacy of a complex medical regimen for each patient. Cost-effectiveness was seen as relevant but not a core consideration, although patient representatives agreed with the prescribing of generic medications where possible.

### RAND/UCLA Appropriateness Method

In stage one, the 160 indicators identified from the systematic review were presented to the panel for consideration and rating. In the stage two face-to-face meeting, panel discussions led to the introduction of 18 re-worded or new indicators, giving a total pool of 178 indicators for consideration. There was panel consensus that 134 indicators were of clinical importance (scoring a median rating of 7 or above); for a further 5 indicators, the panel was equivocal as to clinical importance. The panel agreed that 19 indicators were not of clinical importance, and they did not reach consensus about the importance of 20 indicators. There was a notable lack of prioritisation of, and consensus around, indicators relating to cost-effectiveness, and all indicators in this group were eliminated at this stage.

The remaining 134 indicators represented 17 categories, from which we derived 29 sub-categories. Application of the agreed decision rules led to a final listing of 12 indicators (Table 2 and Box 4), which, in consultation with the panel, were re-worded to be consistent in the use of terminology (e.g. ‘drug’ rather than ‘medication’), grammar (e.g. statements rather than questions) and positive-versus-negative framing. These came from nine previously developed indicator lists (one indicator from each of seven existing lists [14, 24–29] and two indicators each from a further two lists [30, 31]). The panel introduced one entirely new indicator during stage two discussions. The final suggested list was circulated to the panel members for final comment and agreement; minor rewording was suggested and agreed for three indicators as a result (indicators 2, 7, and 11), most notably the insertion of ‘hepatic status’ into indicator 7 (the drug as currently prescribed is not likely to be sub-therapeutic or toxic, based on the dose, route and dosing interval for the age, renal and hepatic status of the patient).

**Table 2:** Origination, panel rating and wording of final agreed indicators

Indicator group	Original indicator wording	Source	Method of development	Revised wording suggested in stage two	Median score	Final revised wording agreed with panel
Adherence	Does the patient adhere to his/her medication schedule?	Drenth-van Maanen, 2009 [24]	- Literature based - Research team - Developed by the research team on basis of literature review		8.5	The patient adheres to the drug schedule
Adverse effect	If a [type A/B] drug reaction occurs, there are details given of the reaction and recommended future monitoring in the patient medical record	Tully, 2005 [30]	- Literature based - Nominal Group Technique (NGT) - Domains of appropriate prescribing established using NGT - Indicators based on this and previously published indicators; refined through cyclical operationalisation in UK hospital setting		9	If an adverse drug reaction occurs, there are details given of the reaction and recommended future monitoring in the medical record
Alternatives to current therapy	Non-pharmacological	Lenaerts, 2013 [25]	- Not stated - No details in source of how indicators were developed	Are there non-pharmacological alternatives?	8.5	There are no effective non-pharmacological alternatives available
Clinical response	Is the drug effective for this indication?	Lara, 2012 [26]	- Literature based - Expert panel - Developed from literature review and a two-stage consensus process with panel of 11 multidisciplinary experts		8.5	The drug is effective in this patient for this indication
Interaction	Are there clinically significant drug–drug interactions?	Hanlon, 1992 [14]	- Literature based - Expert review - Developed from literature review; clinical pharmacist and internist-geriatrician identified key elements to derive criteria		9	There are no clinically significant drug–drug interactions (including duplication of therapy)
Complexity of medication	Could the drug regimen be simplified?	Newton, 1994 [31]	- Expert panel - Two geriatric internists discussed medications of four elderly patients; further internist recorded implicit rules used - Initial algorithm developed and reviewed by panel of 5 internists; final algorithm tested in a hospital outpatient clinic		9	The drug regimen cannot be simplified
Compliance with guidance	Medication selections are consistent with established clinical practice guidelines	Bergman-Evans, 2006 [27]	- Literature based - Research team - Developed by the research team on basis of literature review/evidence synthesis of previously published indicators		8	Drug selection is consistent with established clinical practice
Adequate directions	Is the patient/caregiver unclear about the medication regimen?	Newton, 1994 [31]	- Expert panel - Two geriatric internists discussed medications of four elderly patients; further internist recorded implicit rules used - Initial algorithm developed and reviewed by panel of five internists; final algorithm tested in a hospital outpatient clinic	Is the patient/caregiver clear about the medication regimen?	9	The patient/caregiver is clear about the medication regimen
Contraindication	If the drug is contraindicated, the prescriber gives a valid reason	Cantrill, 1998 [28]	- Nominal group technique - Delphi survey - NGT (panel of nine multidisciplinary experts) convened to derive potential indicators - Two-round Delphi (100 GPs and 100 community pharmacists) used to assess face validity and content validity and develop consensus		8	If the drug is contraindicated, the prescriber gives a valid reason
Indication available	The indication for the drug is recorded in the discharge summary	Tully, 2005 [30]	- Literature based - NGT - Domains of appropriate prescribing established using NGT - Indicators based on this and previously published indicators; refined through cyclical operationalisation in UK hospital setting		8.5	The indication for the drug is recorded in the medical record
Review	The drug treatment is reviewed by an appropriate clinician at least once per year in accordance with best clinical practice	NEW	- Expert panel - Proposed by expert panel during stage 2 discussions		9	The drug treatment is reviewed by an appropriate clinician at least once per year, or more frequently if in accordance with best clinical practice
Dose/route/formulation/frequency	Is the drug as currently given likely to be sub-therapeutic or toxic, based on the dose, route and dosing interval for the age and renal status of the patient?	Hamdy, 1995 [29]	- Research team - Developed by the research team, based on guidelines from drug-regimen review criteria used by consultant pharmacists		8	The drug as currently prescribed is not likely to be sub-therapeutic or toxic, based on the dose, route and dosing interval for the age, renal and hepatic status of the patient

## Discussion

We have identified a set of 12 indicators of prescribing appropriateness suitable for use in the context of a patient with polypharmacy. We are currently operationalising these indicators for use in clinical practice and informatics systems, with the aim of facilitating risk stratification, and the targeting and monitoring of polypharmacy interventions.

Our review identified a proliferation of implicit indicators of inappropriate prescribing; our final set of 12 indicators of polypharmacy appropriateness originated from nine different existing measures, including the influential Medication Appropriateness Index (MAI) [14, 32]. The MAI is comprised of 10 questions, completed by a pharmacist or physician in order to assess the appropriateness of a drug. It has been widely used as an outcome measure in randomised trials of interventions to improve prescribing; however, its authors specifically state that, whilst it is suited to identifying instances of potentially inappropriate prescribing, it was not developed to identify sub-optimal prescribing in the context of polypharmacy [32]. Whilst only one of our indicators was directly derived from those within the MAI, it is important to note that our final list overlaps with the MAI in many ways, suggesting both that a number of core constructs of inappropriate polypharmacy (including indication, effectiveness and interactions) were captured well by this measure, and that subsequent measures of inappropriate prescribing may have been replicating much of the original MAI work.

However, areas of clinical importance not captured by the MAI were also selected by our expert panel, reflecting our particular focus on the context of multiple medications. These included patient adherence, the complexity of the medication regimen and the availability of non-pharmacological alternatives. Determinants of patient adherence are complex; specific factors, including frequent dosing, longer duration of treatment and the presence of adverse effects, may all decrease adherence [33]. Medication regimen complexity is negatively associated with levels of adherence in patients, and medications become increasingly complex with increasing levels of polypharmacy [34]. Increased toxicity as a consequence of multiple medicines [35] may be reduced by using other treatment options. Lifestyle measures and non-pharmacological treatments are key alternative therapeutic options for a range of clinical conditions commonly found in patients with polypharmacy, including depression and cardiovascular risk management [36, 37].

The importance of a regular full review of prescribed medications was formulated as a new indicator specifically following panel discussion. Systematically conducted medication reviews have been shown to be of particular importance to patients with polypharmacy [38] and have been recommended for those over 75 taking multiple drugs. Not conducting regular medication reviews, particularly for those prescribed multiple drugs, places patients at greater risk of potential harm [39]. However, current evidence is limited on the clinical effectiveness of systematically conducted medication review for reducing the suboptimal use of medicines and improving patient-reported outcomes [40].

Cost-effectiveness was included in a number of identified instruments, including the MAI, but discarded by our panel. Their view was that cost-effectiveness in prescribing (typically defined as ensuring that medication is both clinically and economically appropriate for the conditions [41]) was not, in itself, a marker of polypharmacy appropriateness; instead, they viewed cost-effectiveness as a potentially positive consequence of patient-centred medicines optimisation. Our patient panel, too, echoed this opinion, agreeing simply that the prescribing of generic drugs, when available and when cheaper than branded products, was a sensible approach.

A number of strengths and limitations of this study are worth acknowledging. We have conducted a large-scale systematic review, supported by an experienced medical librarian, and followed a well-established consensus method with a diverse expert panel. As polypharmacy is relevant to all ages, we placed no restrictions on age in our analyses in order to ensure the generalisability of the findings. To locate all potentially relevant indicators, we used a high sensitivity, low precision search strategy; we acknowledge the subsequent high citation screening workload (with only 10% double screening) may have therefore resulted in important relevant citations being missed. Most of the indicators located, reviewed and evaluated for clinical importance had previously been subject to robust development processes, increasing our confidence in their face and content validity. However, we note that it is possible we missed additional relevant indicators if they were not readily identifiable as implicit measures of appropriate prescribing or polypharmacy. Whilst we recruited a wide range of experts to the panel, they may not be representative of all healthcare professionals involved in caring for patients with polypharmacy. The lack of international perspectives on the panel, which was convened only from UK participants, may reduce the applicability of our findings to other contexts. Following our stage two panel meeting, we developed and applied additional decision rules to produce a non-duplicative and coherent indicator list; whilst this was done in consultation with the panel, we acknowledge that other research teams may have made different decisions about which indicators to retain. Additional work will be necessary to explore the acceptability and operationalisation of the chosen indicators within clinical systems, and to ascertain their utility for risk stratification, targeting and monitoring of polypharmacy interventions. This work will additionally consider issues such as whether indicators should be weighted in an assessment of polypharmacy appropriateness. Finally, we note that this approach does not by itself facilitate the inclusion of patient perspectives of polypharmacy appropriateness. Whilst our proposed measure, when operationalised, may highlight patients where there are clinical concerns about medication regimen, it cannot offer a holistic assessment of the appropriateness of the regimen, where the patients’ perspectives must be central to any decisions about care.

## Conclusions

This study has integrated and adapted existing indicators of appropriate prescribing, and introduced a new one, to produce a short, yet comprehensive list of 12 indicators suitable to the assessment of prescribing appropriateness in a patient with polypharmacy. Use of these indicators in clinical practice and informatics systems is dependent on their operationalisation, and their utility (e.g. risk stratification, targeting and monitoring polypharmacy interventions) requires subsequent evaluation.

## Box 1 MEDLINE search strategy

(exp Inappropriate prescribing/ or exp. polypharmacy/ or exp. medication errors/ or exp. Potentially Inappropriate Medication List/ or (polypharmacy or underprescrib* or under-prescrib* or over-prescrib* or mis-prescrib* or overprescrib* or misprescrib* or (beer* adj criteri*) or (pim adj list*)).ti,ab. or ((prescrib* or prescript* or medicat* or medicin* or drug* or pharm*) adj2 (sub-optimal or suboptimal or optim* or appropriat* or inappropriat* or unaccept* or accept* or underus* or under-us* or over-us* or overus* or underutili* or under-utili* or malpractice* or safe* or unsafe* or danger* or error* or mistak* or (adverse* adj (event* or effect* or react*)) or harm* or omiss* or omit* or problem*)).ti,ab.) AND (((exp “Surveys and Questionnaires”/ or exp. guideline/ or exp. quality assurance, health care/) and ((updat* or develop* or valid* or creat* or design* or consensus* or Delphi or rand* or reliab* or interrat* or inter-rate* or (inter adj rate*) or (appropriate* adj method*)).ti,ab.)) or (((score* or index* or scale* or survey* or questionnaire* or instrument* or outcome* or tool* or indicat* or measur* or screen* or criteri* or (quality adj2 assur*) or (patient adj2 experience*)) adj4 (updat* or develop* or valid* or creat* or design* or consensus* or Delphi or rand* or reliab* or interrat* or inter-rate* or (inter adj rate*) or (appropriate* adj method*))).ti,ab.))

## Box 2 MEDLINE search strategy for review of reviews

1. ((exp Inappropriate prescribing/ or exp. polypharmacy/ or exp. medication errors/ or exp. Potentially Inappropriate Medication List/ or (polypharmacy or underprescrib* or under-prescrib* or over-prescrib* or mis-prescrib* or overprescrib* or misprescrib* or (beer* adj criteri*) or (pim adj list*)).ti,ab.) Or ((prescrib* or prescript* or medicat* or medicin* or drug* or pharm*) adj2 (sub-optimal or suboptimal or optim* or appropriat* or inappropriat* or unaccept* or accept* or underus* or under-us* or over-us* or overus* or underutili* or under-utili* or malpractice* or safe* or unsafe* or danger* or error* or mistak* or (adverse* adj (event* or effect* or react*)) or harm* or omiss* or omit* or problem*)).ti,ab.) And ((exp “Surveys and Questionnaires”/ or exp. guideline/ or exp. quality assurance, health care/) or (score* or index* or scale* or survey* or questionnaire* or instrument* or outcome* or tool* or indicat* or measur* or screen* or criteri* or (quality adj2 assur*) or (patient adj2 experience*)).ti,ab.) and ((updat* or develop* or valid* or creat* or design* or consensus* or Delphi or rand* or reliab* or interrat* or inter-rate* or (inter adj rate*) or (appropriate* adj method*)).ti,ab.)

2. ((exp Inappropriate prescribing/ or exp. polypharmacy/ or exp. medication errors/ or exp. Potentially Inappropriate Medication List/ or (polypharmacy or underprescrib* or under-prescrib* or over-prescrib* or mis-prescrib* or overprescrib* or misprescrib* or (beer* adj criteri*) or (pim adj list*)).ti,ab.) Or ((prescrib* or prescript* or medicat* or medicin* or drug* or pharm*) adj2 (sub-optimal or suboptimal or optim* or appropriat* or inappropriat* or unaccept* or accept* or underus* or under-us* or over-us* or overus* or underutili* or under-utili* or malpractice* or safe* or unsafe* or danger* or error* or mistak* or (adverse* adj (event* or effect* or react*)) or harm* or omiss* or omit* or problem*)).ti,ab.) And ((exp “Surveys and Questionnaires”/ or exp. guideline/ or exp. quality assurance, health care/) or (score* or index* or scale* or survey* or questionnaire* or instrument* or outcome* or tool* or indicat* or measur* or screen* or criteri* or (quality adj2 assur*) or (patient adj2 experience*)).ti,ab.) and ((updat* or develop* or valid* or creat* or design* or consensus* or Delphi or rand* or reliab* or interrat* or inter-rate* or (inter adj rate*) or (appropriate* adj method*)).ti,ab.) AND ((systematic adj (review$1 or overview$1)).tw.)

2 not 1

## Box 3 Prescribing indicator categories, derived from content analysis of identified indicators

1. Adherence

2. Adverse drug reactions

3. Drug-drug interactions

4. Medication review

5. Contraindication (drug-disease interactions)

6. Alternatives to current therapy

7. Clinical efficacy

8. Complexity of medication

9. Compliance with guidance

10. Cost-effectiveness

11. Directions

12. Dosage/duration

13. Duplication

14. Other inappropriate prescribing

15. Indication

16. Under-prescribing

17. Specific safety issues

18. General indicators

## Box 4 Final indicators as agreed by expert panel

Implicit indicators of polypharmacy appropriateness

For this specific drug:

1. The indication for the drug is recorded in the medical record

2. There are no effective non-pharmacological alternatives available

3. Drug selection is consistent with established clinical practice

4. There are no clinically significant drug-drug interactions (including duplication of therapy)

5. If the drug is contraindicated, the prescriber gives a valid reason

6. The drug is effective in this patient for this indication

7. The drug, as currently prescribed, is not likely to be sub-therapeutic or toxic, based on the dose, route and dosing interval for the age, renal and hepatic status of the patient

8. The drug regimen cannot be simplified

9. The patient/caregiver is clear about the drug regimen

10. The patient adheres to the drug schedule

11. The drug treatment is reviewed by an appropriate clinician at least once per year, or more frequently if in accordance with best clinical practice

12. If an adverse drug reaction occurs, there are details given of the reaction and recommended future monitoring in the medical record

### Additional file

Additional file 1: Table of included indicators, by category grouping. (DOCX 67 kb)

## Appendix 1

**Table 3:** Table of included studies

Authors and year	Country	Setting	Development of indicators	Name of instrument	Number of indicators	Implicit only or mixed (explicit/implicit) indicators	Example indicator
Basger et al., 2008 [18]	Australia	Primary care	Literature review and expert discussions to develop indicators	Australian prescribing indicators for commonly occurring conditions in patients aged > 65 years	48	Mixed	Patient has no significant medication interactions (agreement between two medication interaction databases)
Basger et al., 2012 [19]	Australia	Primary care	Literature review and expert discussions to develop indicators RAND/UCLA Appropriateness Method to refine indicators	Validated prescribing appropriateness criteria for older Australians (≥ 65 years) for commonly used medications and medical condition	41	Mixed	Patient has no clinically significant medication interactions (agreement between two medication interaction databases)
Bergman-Evans, 2006 [27]	USA	Not confined to one setting	Literature review to develop indicators	Medication Management Outcomes Monitor	18	Implicit	Medications prescribed match established diagnosis
Buetow et al., 1996 [42]	UK	Primary care	Nominal group technique to develop indicators Indicators applied to studies located through systematic review	Dimensions and indicators of prescribing appropriateness	19	Implicit	The formulation and route and method of delivery are designed to maximise compliance for an individual patient
Cantrill et al., 1998 [28]	UK	Primary care	Nominal Group Technique to develop indicators Delphi method to assess validity of indicators	Indicators of appropriateness of prescribing	9	Mixed	If a potentially hazardous drug–drug combination is prescribed, the prescriber shows knowledge of the hazard
Caughey et al., 2014 [43]	Australia	Primary care	Literature review and review of clinical indicators to identify existing indicators and develop new ones Modified RAND appropriateness method to assess validity of indicators	Australian medication-related indicators of potentially preventable hospitalisations	29	Mixed	Use of two or more agents with anticholinergic activity OR use of an agent with high anticholinergic activity
Drenth-van Maanen et al., 2009 [24]	The Netherlands	Primary care	No detail on how indicators developed	Prescribing Optimization Method	6	Implicit	Which adverse effects are present?
Fried et al., 2016 [44]	USA	Not confined to one setting	Literature review and expert discussions to develop indicators Modified Delphi method to refine indicators	Strategies for addressing problems with medication regimens	10	Mixed	It is reasonable to undertake dose reduction or discontinuation of medications associated with both benefits and side effects if the patient views the side effects as more important than the benefits
Gazarian et al., 2006 [45]	Australia	Not confined to one setting	Expert working party using consensus-based approaches to develop decision algorithm	Assessing appropriateness of off-label medicines use	Not available – decision algorithm with accompanying explanatory notes	Implicit	Will this medicine be used according to a registered indication, age, dose and route?
Hamdy et al., 1995 [29]	USA	Care homes	Literature review to develop indicators	Criteria for medication profile review	5	Implicit	Are any significant drug–drug or drug–disease interactions present?
Hanlon et al., 1992 [14]	USA	Internal medicine	Literature review and expert discussions to develop indicators	Medication Appropriateness Index	10	Implicit	Is the dosage correct?
Hassan et al., 2010 [46]	Malaysia	Not confined to one setting	Literature review and expert discussions to develop indicators Modified Delphi method to assessing validity of indicators	Prescription Quality Index	22	Implicit	Is there unnecessary duplication with other drug(s)?
Johnson et al., 1995 [47]	USA	Pharmacy	Literature review and expert discussions to develop indicators	–	10	Implicit	Interaction: drug–drug
Lara et al., 2012 [26]	Spain	Not confined to one setting	Literature review to identify indicators Delphi method to refine indicators	–	12	Implicit	Is there a lack of diagnoses or symptoms recorded in the medical history that do not have drug treatments but could have it?
Lenaerts et al., 2013 [25]	Belgium	Primary care	No detail on how indicators developed	Appropriate Medication for Older people-tool	8	Implicit	Are dosage and dosage form adapted to the patient?
Newton et al., 1994 [31]	USA	Primary care	Expert discussions to develop indicators	The Geriatric Medication Evaluation Algorithm	10	Implicit	Is the patient/caregiver unclear about the medication regimen?
O’Mahoney et al., 2014 [48]	Europe	Not confined to one setting	Literature review and expert consultation to review existing indicators and propose new ones Two-round Delphi method to refine and validate indicators	STOPP/START	114 (80 STOPP; 34 START)	Mixed	Any drug prescribed without an evidence-based clinical indication
Stange et al., 2010 [49]	Germany	Not confined to one setting	Forward and backward translation of the English version of the MCRI	Medication Regimen Complexity Index – German	1	Implicit	Not available: three sections (Section A: dosage forms; Section B: dosage frequency; Section C: additional instructions) to compute a score indicating the complexity of a given pharmacotherapeutic regimen
Tommelein et al., 2015 [50]	Belgium	Primary care	Literature review and two-round RAND/UCLA Appropriateness method to develop indicators	Ghent Older People’s Prescriptions community Pharmacy Screening (GheOP^3^S) tool	83	Mixed	Polypharmacy patients (chronically taking five or more drugs) were not questioned about whether a clear medication scheme was available to them
Tully et al., 2005 [30]	UK	Secondary care	Literature review and expert discussions to develop indicators Pre- and pilot-testing on patient records, and expert panel, to assess validity of indicators	Appropriateness of long-term prescribing commenced in hospital practice	14	Implicit	Hazardous drug–drug combination
van Dijk et al., 2003 [51]	The Netherlands	Primary care	Does not state how indicators developed	Evaluation of drug use in nursing homes	6	Mixed	More than one drug from same drug class
Winslade et al., 1997 [52]	Canada	Pharmacy	Expert discussions and application in practice to revise two previous sets of indicators	Pharmacist management of drug-related problems	8	Implicit	The patient is taking/receiving a drug for which there is no valid indication
