# Supplementary material for: Developing a measure of polypharmacy appropriateness in primary care: systematic review and expert consensus study
Source: BMC Med. 2018 Jun 13;16:91. doi: 10.1186/s12916-018-1078-7 (PMC5998565; doi:10.1186/s12916-018-1078-7)
Supplement: Supplementary file 1 — Table of included indicators, by category grouping. (DOCX 67 kb) [file 12916_2018_1078_MOESM1_ESM.docx]

**Additional file 1:** Table of included indicators, by category grouping

| **Adherence** | | | |
| --- | --- | --- | --- |
| **Unique indicator number** | **Indicator** | **Source** | **Method of development** |
| 84 | Does the Patient Adhere to His/Her Medication Schedule? | Prescribing Optimization Method for Improving Prescribing in Elderly Patients Receiving Polypharmacy Results of Application to Case Histories by General Practitioners (Drenth-van Maanen et al 2009) | **Research team; Literature based:** These indicators (originally listed in the form of questions with short evidence overview) were developed by the research team; the evidence overview is stated to be based on ‘available literature.’ |
| 5 | Evidence of failure to refill medication, as obtained from electronic medical record, pharmacist, or other available sources | A Delphi process to address medication  appropriateness for older persons with  multiple chronic conditions (Fried et al 2016) | **Delphi method; literature based; research team:** These indicators were developed using a modified Delphi technique. Included ‘time-zero’ meeting (from RAND/UCLA appropriateness method) and core research team generated a candidate set of items for the panel to consider based on the results of the time zero meeting and literature review. The nine panellists represented the disciplines of nursing, medicine and pharmacy. |
| 334 | No indications of noncompliance from client self report or history/physical findings. | Evidence-Based Guideline  Improving Medication Management  for Older Adult Clients (Bergman-Evans, 2006) | **Literature based:** Not stated how these indicators were developed: paper is a review/evidence synthesis of other published indicators. Indicators not stated to have been subject to any consensus development. |
| 322 | Non-compliance | Measuring the Impact of Patient Counseling in the Outpatient Pharmacy Setting: Development and Implementation of the Counseling Models for the Kaiser Permanente AJSC Patient Consultation Study (Johnson et al 1995) | **Literature based; Expert panel:** A multidisciplinary team developed screening criteria for determining high-risk patients. The team reviewed the literature and internal data concerning drug-related problems associated with a high likelihood of hospital admission. From this information, three screening criteria were developed. |
| 1 | Patient and/or caregiver self-report of non-adherence | A Delphi process to address medication  appropriateness for older persons with  multiple chronic conditions (Fried et al 2016) | **Delphi method; literature based; research team:** These indicators were developed using a modified Delphi technique. Included ‘time-zero’ meeting (from RAND/UCLA appropriateness method) and core research team generated a candidate set of items for the panel to consider based on the results of the time zero meeting and literature review. The nine panellists represented the disciplines of nursing, medicine and pharmacy. |
| 303 | The patient is not taking/receiving the prescribed drug appropriately. | Pharmacist's Management of Drug-Related Problems: A Tool for Teaching and Providing Pharmaceutical Care (Winslade et al 1997) | **Expert panel**: The development of these indicators was initially based on two existing pharmaceutical care tools, which were modified significantly through practice models and experience. |
| **Adverse drug reactions** | | | |
| **Indicator Number** | **Indicator** | **Source** | **Method of development** |
| 98 | Adverse drug reactions: Unexpected drug reactions (type B) are investigated and recorded. Prescribing of the drug is continued only with caution and monitoring | Prevalence of potentially inappropriate long term prescribing in general practice in the United  Kingdom, 1980–95: systematic literature review (Buetow et al 1996) | **Expert panel:** A panel of 10-experts used professional opinion to suggest consensus-based criteria. The discussions were recorded and transcribed. These were used by the research team to develop 19 implicit indicators covering five dimensions: indication, choice of drug, drug administration, communication, and review. |
| 325 | Allergy/Sensitivity | Measuring the Impact of Patient Counseling in the Outpatient Pharmacy Setting: Development and Implementation of the Counseling Models for the Kaiser Permanente AJSC Patient Consultation Study (Johnson et al 1995) | **Literature based; Expert panel:** A multidisciplinary team developed screening criteria for determining high-risk patients. The team reviewed the literature and internal data concerning drug-related problems associated with a high likelihood of hospital admission. From this information, three screening criteria were developed. |
| 31 | Are there side-effects reported? | Appropriate prescribing for older people: a new tool for the general practitioner (Lenaerts et al 2013). | **Unknown:** There is no detail in the original source of how these indicators were developed. |
| 308 | Could this drug be aggravating underlying conditions or be causing subtle atypical side effects? | The Geriatric Medication Algorithm: A Pilot Study (Newton et al. 1994) | **Expert panel:** Two geriatric internists discussed medications of four elderly patients and a further internist recorded their implicit rules. An initial algorithm was developed and reviewed by a panel of five further internists. Final algorithm was tested in a resident outpatient clinic of a teaching hospital. |
| 64 | Does the patient experience any adverse drug reaction? | Development and validation of a new Prescription Quality Index (Hassan et al 2009) | **Literature based; Expert panel; Delphi Method:** Initial items were developed from an extensive review of the literature and discussion with experts. The first version of the PQI was piloted, and modified. Validity was examined using the Delphi Method in two separate surveys. |
| 52 | If a type A drug reaction occurs, there are details given of the reaction and recommended future monitoring in the patient medical record | Development and face validity of explicit indicators of appropriateness of long  term prescribing (Tully et al 2005) | **Literature based, research team, Nominal Group Technique:** Version 1 of the indicators was developed from both the earlier work of the authors and previously published indicators. Domains of appropriate prescribing had been developed by the authors, using the nominal group technique. Indicators were then refined for the hospital setting in the UK, operationalised using patient records by a member of the research team and a clinical pharmacist, refined further, operationalised with a further sample of patient records and refined again, before being subjected to face validity testing. |
| 53 | If a type B drug reaction occurs, the prescriber gives details of the reaction and recommended action in both the inpatient record and the discharge summary. | Development and face validity of explicit indicators of appropriateness of long  term prescribing (Tully et al 2005) | **Literature based, research team, Nominal Group Technique:** Version 1 of the indicators was developed from both the earlier work of the authors and previously published indicators. Domains of appropriate prescribing had been developed by the authors, using the nominal group technique. Indicators were then refined for the hospital setting in the UK, operationalised using patient records by a member of the research team and a clinical pharmacist, refined further, operationalised with a further sample of patient records and refined again, before being subjected to face validity testing. |
| 328 | Is the patient receiving one or more drugs that may have been prescribed in response to an adverse drug reaction? If so, can the drug associated with the adverse drug reaction be withdrawn or substituted with another agent less likely to be associated with such a reaction? | Reducing Polypharmacy in Extended care (Hamdy et al. 1995) | **Literature based:** These indicators were developed using guidelines from drug-regimen review criteria, used by consultant pharmacists. No other detail is provided and these were not subject to a consensus process. |
| 346 | No hospitalizations or emergency department visits related to adverse drug reactions. | Evidence-Based Guideline  Improving Medication Management  for Older Adult Clients (Bergman-Evans, 2006) | **Literature based:** Not stated how these indicators were developed: paper is a review/evidence synthesis of other published indicators. Indicators not stated to have been subject to any consensus development. |
| 344 | Patient reports no adverse drug reactions or side effects. | Evidence-Based Guideline  Improving Medication Management  for Older Adult Clients (Bergman-Evans, 2006) | **Literature based:** Not stated how these indicators were developed: paper is a review/evidence synthesis of other published indicators. Indicators not stated to have been subject to any consensus development. |
| 14 | Patient self-report of a significant or serious adverse effect of a medication | A Delphi process to address medication  appropriateness for older persons with  multiple chronic conditions (Fried et al 2016) | **Delphi method; literature based; research team:** These indicators were developed using a modified Delphi technique. Included ‘time-zero’ meeting (from RAND/UCLA appropriateness method) and core research team generated a candidate set of items for the panel to consider based on the results of the time zero meeting and literature review. The nine panellists represented the disciplines of nursing, medicine and pharmacy. |
| 320 | Side effects/Toxicity | Measuring the Impact of Patient Counseling in the Outpatient Pharmacy Setting: Development and Implementation of the Counseling Models for the Kaiser Permanente AJSC Patient Consultation Study (Johnson et al 1995) | **Literature based; Expert panel:** A multidisciplinary team developed screening criteria for determining high-risk patients. The team reviewed the literature and internal data concerning drug-related problems associated with a high likelihood of hospital admission. From this information, three screening criteria were developed. |
| 345 | The medication list does not include drugs prescribed to treat side effects or adverse reactions. | Evidence-Based Guideline  Improving Medication Management  for Older Adult Clients (Bergman-Evans, 2006) | **Literature based:** Not stated how these indicators were developed: paper is a review/evidence synthesis of other published indicators. Indicators not stated to have been subject to any consensus development. |
| 304 | The patient is experiencing an adverse drug reaction (defined as not dose related). | Pharmacist's Management of Drug-Related Problems: A Tool for Teaching and Providing Pharmaceutical Care (Winslade et al 1997) | **Expert panel**: The development of these indicators was initially based on two existing pharmaceutical care tools, which were modified significantly through practice models and experience. |
| 45 | If the drug is part of a questionable high-risk therapeutic combination, the prescriber gives a reason in the discharge summary. | Development and face validity of explicit indicators of appropriateness of long  term prescribing (Tully et al 2005) | **Literature based, research team, Nominal Group Technique:** Version 1 of the indicators was developed from both the earlier work of the authors and previously published indicators. Domains of appropriate prescribing had been developed by the authors, using the nominal group technique. Indicators were then refined for the hospital setting in the UK, operationalised using patient records by a member of the research team and a clinical pharmacist, refined further, operationalised with a further sample of patient records and refined again, before being subjected to face validity testing. |
| 307 | Is this a high risk drug? | The Geriatric Medication Algorithm: A Pilot Study (Newton et al. 1994) | **Expert panel:** Two geriatric internists discussed medications of four elderly patients and a further internist recorded their implicit rules. An initial algorithm was developed and reviewed by a panel of five further internists. Final algorithm was tested in a resident outpatient clinic of a teaching hospital. |
| **Alternatives to current therapies** | | | |
| **Indicator Number** | **Indicator** | **Source** | **Method of development** |
| 37 | Are there alternatives | Appropriate prescribing for older people: a new tool for the general practitioner (Lenaerts et al 2013). | **Unknown:** There is no detail in the original source of how these indicators were developed. |
| 310 | Is there a less toxic drug? | The Geriatric Medication Algorithm: A Pilot Study (Newton et al. 1994) | **Expert panel:** Two geriatric internists discussed medications of four elderly patients and a further internist recorded their implicit rules. An initial algorithm was developed and reviewed by a panel of five further internists. Final algorithm was tested in a resident outpatient clinic of a teaching hospital. |
| 38 | Non pharmacological | Appropriate prescribing for older people: a new tool for the general practitioner (Lenaerts et al 2013). | **Unknown:** There is no detail in the original source of how these indicators were developed. |
| **Clinical efficacy** | | | |
| **Indicator Number** | **Indicator** | **Source** | **Method of development** |
| 91 | Drugs of limited value: Drugs of limited value are avoided or the reason for their use is stated in the medical records | Prevalence of potentially inappropriate long term prescribing in general practice in the United  Kingdom, 1980–95: systematic literature review (Buetow et al 1996) | **Expert panel:** A panel of 10-experts used professional opinion to suggest consensus-based criteria. The discussions were recorded and transcribed. These were used by the research team to develop 19 implicit indicators covering five dimensions: indication, choice of drug, drug administration, communication, and review. |
| 109 | Effectiveness: The prescription produces a beneficial outcome in the patient | Prevalence of potentially inappropriate long term prescribing in general practice in the United  Kingdom, 1980–95: systematic literature review (Buetow et al 1996) | **Expert panel:** A panel of 10-experts used professional opinion to suggest consensus-based criteria. The discussions were recorded and transcribed. These were used by the research team to develop 19 implicit indicators covering five dimensions: indication, choice of drug, drug administration, communication, and review. |
| 222 | Is the drug effective for this indication? | Selection of tools for reconciliation, compliance and appropriateness of treatment in patients with multiple chronic conditions (Lara et al 2012) | **Literature based; Expert panel:** Indicators were developed from a literature review (including grey literature). A panel of 11-experts of a multidisciplinary background subjected the indicators to a two round consensus process. However, it was unclear if this occurred face-to face or remotely. |
| 18 | Is the medication effective for the condition? | A method for assessing drug therapy appropriateness [MAI]. (Hanlon et al 1992) | **Literature based; Expert panel:** These indicators were developed through a review of literature related to drug-related problems. A Clinical pharmacist and an Internist-geriatrician identified key elements based on this literature and experience and created the MAI criteria. |
| 59 | Is the usage of the drug for the indication supported by evidence? | Development and validation of a new Prescription Quality Index (Hassan et al 2009) | **Literature based; Expert panel; Delphi Method:** Initial items were developed from an extensive review of the literature and discussion with experts. The first version of the PQI was piloted, and modified. Validity was examined using the Delphi Method in two separate surveys. |
| 30 | Is there response on the treatment? | Appropriate prescribing for older people: a new tool for the general practitioner (Lenaerts et al 2013). | **Unknown:** There is no detail in the original source of how these indicators were developed. |
| 15 | Patient self-report that the medication regiment is not achieving the desired outcome | A Delphi process to address medication  appropriateness for older persons with  multiple chronic conditions (Fried et al 2016) | **Delphi method; literature based; research team:** These indicators were developed using a modified Delphi technique. Included ‘time-zero’ meeting (from RAND/UCLA appropriateness method) and core research team generated a candidate set of items for the panel to consider based on the results of the time zero meeting and literature review. The nine panellists represented the disciplines of nursing, medicine and pharmacy. |
| 48 | The drug is considered to be efficacious for the condition, or the prescriber gives a reason for prescribing it in the discharge summary. | Development and face validity of explicit indicators of appropriateness of long  term prescribing (Tully et al 2005) | **Literature based, research team, Nominal Group Technique:** Version 1 of the indicators was developed from both the earlier work of the authors and previously published indicators. Domains of appropriate prescribing had been developed by the authors, using the nominal group technique. Indicators were then refined for the hospital setting in the UK, operationalised using patient records by a member of the research team and a clinical pharmacist, refined further, operationalised with a further sample of patient records and refined again, before being subjected to face validity testing. |
| 71 | The reason for prescribing a drug of limited value is recorded and valid | Indicators of the appropriateness of long term  prescribing in general practice in the United  Kingdom: consensus development, face and  content validity, feasibility, and reliability (Cantrill et al 1998) | **Nominal Group Technique:** 9-Panel member comprising multi-disciplinary background. Each indicator was discussed, and the list refined. A Delphi technique (two rounds) was used to assess face validity and content validity and to develop consensus. A minimum sample of 100 respondents per discipline was required for the survey. |
| 16 | Benefits associated with medications prescribed for prevention, expressed in terms of absolute risk reductions, not sufficiently large from the patient’s perspective | A Delphi process to address medication  appropriateness for older persons with  multiple chronic conditions (Fried et al 2016) | **Delphi method; literature based; research team:** These indicators were developed using a modified Delphi technique. Included ‘time-zero’ meeting (from RAND/UCLA appropriateness method) and core research team generated a candidate set of items for the panel to consider based on the results of the time zero meeting and literature review. The nine panellists represented the disciplines of nursing, medicine and pharmacy. |
| **Complexity of medication** | | | |
| **Indicator Number** | **Indicator** | **Source** | **Method of development** |
| 313 | Could the drug regimen be simplified? | The Geriatric Medication Algorithm: A Pilot Study (Newton et al. 1994) | **Expert panel:** Two geriatric internists discussed medications of four elderly patients and a further internist recorded their implicit rules. An initial algorithm was developed and reviewed by a panel of five further internists. Final algorithm was tested in a resident outpatient clinic of a teaching hospital. |
| 337 | Medications are prescribed no more than twice daily. | Evidence-Based Guideline  Improving Medication Management  for Older Adult Clients (Bergman-Evans, 2006) | **Literature based:** Not stated how these indicators were developed: paper is a review/evidence synthesis of other published indicators. Indicators not stated to have been subject to any consensus development. |
| 104 | Regimen Communication: The dosing schedule is made as simple as possible (to maximise compliance) | Prevalence of potentially inappropriate long term prescribing in general practice in the United  Kingdom, 1980–95: systematic literature review (Buetow et al 1996) | **Expert panel:** A panel of 10-experts used professional opinion to suggest consensus-based criteria. The discussions were recorded and transcribed. These were used by the research team to develop 19 implicit indicators covering five dimensions: indication, choice of drug, drug administration, communication, and review. |
| **Compliance with guidance** | | | |
| **Indicator Number** | **Indicator** | **Source** | **Method of development** |
| 283 | Assessing appropriateness of off-label medicines use | Off-label use of medicines: consensus recommendations for evaluating appropriateness (Gazarian et al 2006) | **Expert panel:** These indicators are drawn from an algorithm as an accompanying document to help guide decision making around off-label drug use. The panel were members of the New South Wales Therapeutic Advisory Group (NSW  TAG) which is an independent state government-funded organisation that aims to promote QUM in the state’s public hospitals and the wider community through collaboration and consensus. Unclear how the algorithm was developed, although likely as part of the consensus process with the NSW TAG group. |
| 69 | Is the medication available in the formulary or essential drug list? | Development and validation of a new Prescription Quality Index (Hassan et al 2009) | **Literature based; Expert panel; Delphi Method:** Initial items were developed from an extensive review of the literature and discussion with experts. The first version of the PQI was piloted, and modified. Validity was examined using the Delphi Method in two separate surveys. |
| 332 | Medication selections are consistent with established clinical practice guidelines. | Evidence-Based Guideline  Improving Medication Management  for Older Adult Clients (Bergman-Evans, 2006) | **Literature based:** Not stated how these indicators were developed: paper is a review/evidence synthesis of other published indicators. Indicators not stated to have been subject to any consensus development. |
| 292 | Use of drugs outside formulary | Prescribing Indicators for Evaluating Drug Use in Nursing Homes (van Dijk et al 2003) | **Research team:** No mention of indicators being based on literature or subjected to expert consensus. |
| 49 | The drug is in the hospital formulary or the prescriber gives a reason for prescribing it in the patient medical record. | Development and face validity of explicit indicators of appropriateness of long  term prescribing (Tully et al 2005) | **Literature based, research team, Nominal Group Technique:** Version 1 of the indicators was developed from both the earlier work of the authors and previously published indicators. Domains of appropriate prescribing had been developed by the authors, using the nominal group technique. Indicators were then refined for the hospital setting in the UK, operationalised using patient records by a member of the research team and a clinical pharmacist, refined further, operationalised with a further sample of patient records and refined again, before being subjected to face validity testing. |
| **Contraindication (drug-disease interactions)** | | | |
| **Indicator Number** | **Indicator** | **Source** | **Method of development** |
| 23 | Are there clinically significant drug disease/condition interactions? | A method for assessing drug therapy appropriateness [MAI]. (Hanlon et al 1992) | **Literature based; Expert panel:** These indicators were developed through a review of literature related to drug-related problems. A Clinical pharmacist and an Internist-geriatrician identified key elements based on this literature and experience and created the MAI criteria. |
| 63 | Are there clinically significant drug–disease/condition interactions? | Development and validation of a new Prescription Quality Index (Hassan et al 2009) | **Literature based; Expert panel; Delphi Method:** Initial items were developed from an extensive review of the literature and discussion with experts. The first version of the PQI was piloted, and modified. Validity was examined using the Delphi Method in two separate surveys. |
| 285 | Contraindications that can unambiguously be derived from patient’s medication were not added to the electronic patient record | Older patients’ prescriptions screening in the community pharmacy: development of the Ghent Older People’s Prescriptions community Pharmacy Screening (GheOP3S) tool (Tommelein et al 2015) | **Literature based; RAND/UCLA appropriateness method:** It included (i) a round zero meeting (research team), (ii) a literature review, (iii) a first written Delphi round (multi-disciplinary 11-panelist group participated), (iv) a second face-to-face Delphi round based on the first round evaluation (only 8 panellists attended) and (v) finally, a selection of those items considered applicable in the contemporary community pharmacy practice (a panel of seven Belgian community pharmacists were invited to select those items that were applicable in the contemporary community pharmacy practice). |
| 99 | Contraindications: Contraindicated drugs, as recorded in the British National Formulary, are not prescribed unless the prescriber explicitly indicates that the potential benefits outweigh the risks | Prevalence of potentially inappropriate long term prescribing in general practice in the United  Kingdom, 1980–95: systematic literature review (Buetow et al 1996) | **Expert panel:** A panel of 10-experts used professional opinion to suggest consensus-based criteria. The discussions were recorded and transcribed. These were used by the research team to develop 19 implicit indicators covering five dimensions: indication, choice of drug, drug administration, communication, and review. |
| 324 | Drug-disease Interaction | Measuring the Impact of Patient Counseling in the Outpatient Pharmacy Setting: Development and Implementation of the Counseling Models for the Kaiser Permanente AJSC Patient Consultation Study (Johnson et al 1995) | **Literature based; Expert panel:** A multidisciplinary team developed screening criteria for determining high-risk patients. The team reviewed the literature and internal data concerning drug-related problems associated with a high likelihood of hospital admission. From this information, three screening criteria were developed. |
| 55 | If a potentially hazardous drug-disease combination is prescribed, the prescriber gives a reason in the discharge summary | Development and face validity of explicit indicators of appropriateness of long  term prescribing (Tully et al 2005) | **Literature based, research team, Nominal Group Technique:** Version 1 of the indicators was developed from both the earlier work of the authors and previously published indicators. Domains of appropriate prescribing had been developed by the authors, using the nominal group technique. Indicators were then refined for the hospital setting in the UK, operationalised using patient records by a member of the research team and a clinical pharmacist, refined further, operationalised with a further sample of patient records and refined again, before being subjected to face validity testing. |
| 75 | If the drug is contraindicated, the prescriber gives a valid reason | Indicators of the appropriateness of long term  prescribing in general practice in the United  Kingdom: consensus development, face and  content validity, feasibility, and reliability (Cantrill et al 1998) | **Nominal Group Technique:** 9-Panel member comprising multi-disciplinary background. Each indicator was discussed, and the list refined. A Delphi technique (two rounds) was used to assess face validity and content validity and to develop consensus. A minimum sample of 100 respondents per discipline was required for the survey. |
| 228 | Is there a lack of contraindications for this drug? | Selection of tools for reconciliation, compliance and appropriateness of treatment in patients with multiple chronic conditions (Lara et al 2012) | **Literature based; Expert panel:** Indicators were developed from a literature review (including grey literature). A panel of 11-experts of a multidisciplinary background subjected the indicators to a two round consensus process. However, it was unclear if this occurred face-to face or remotely. |
| **Cost-effectiveness** | | | |
| **Indicator Number** | **Indicator** | **Source** | **Method of development** |
| 73 | A generic product is prescribed if one is available | Indicators of the appropriateness of long term  prescribing in general practice in the United  Kingdom: consensus development, face and  content validity, feasibility, and reliability (Cantrill et al 1998) | **Nominal Group Technique:** 9-Panel member comprising multi-disciplinary background. Each indicator was discussed, and the list refined. A Delphi technique (two rounds) was used to assess face validity and content validity and to develop consensus. A minimum sample of 100 respondents per discipline was required for the survey. |
| 72 | Compared with alternative treatments in the same therapeutic class, which are just as safe and effective, the drug prescribed is either one of the cheapest or a valid reason is given for using an alternative | Indicators of the appropriateness of long term  prescribing in general practice in the United  Kingdom: consensus development, face and  content validity, feasibility, and reliability (Cantrill et al 1998) | **Nominal Group Technique:** 9-Panel member comprising multi-disciplinary background. Each indicator was discussed, and the list refined. A Delphi technique (two rounds) was used to assess face validity and content validity and to develop consensus. A minimum sample of 100 respondents per discipline was required for the survey. |
| 94 | Cost effectiveness: The ratio of benefits to costs is better than that for alternative treatments (including non-drug treatments) | Prevalence of potentially inappropriate long term prescribing in general practice in the United  Kingdom, 1980–95: systematic literature review (Buetow et al 1996) | **Expert panel:** A panel of 10-experts used professional opinion to suggest consensus-based criteria. The discussions were recorded and transcribed. These were used by the research team to develop 19 implicit indicators covering five dimensions: indication, choice of drug, drug administration, communication, and review. |
| 93 | Cost minimisation: The drug prescribed is cheaper than alternative treatment(s) but just as safe and effective | Prevalence of potentially inappropriate long term prescribing in general practice in the United  Kingdom, 1980–95: systematic literature review (Buetow et al 1996) | **Expert panel:** A panel of 10-experts used professional opinion to suggest consensus-based criteria. The discussions were recorded and transcribed. These were used by the research team to develop 19 implicit indicators covering five dimensions: indication, choice of drug, drug administration, communication, and review. |
| 333 | Generic and/or least costly appropriate medications are used. | Evidence-Based Guideline  Improving Medication Management  for Older Adult Clients (Bergman-Evans, 2006) | **Literature based:** Not stated how these indicators were developed: paper is a review/evidence synthesis of other published indicators. Indicators not stated to have been subject to any consensus development. |
| 231 | Is it the most cost-effective drug? | Selection of tools for reconciliation, compliance and appropriateness of treatment in patients with multiple chronic conditions (Lara et al 2012) | **Literature based; Expert panel:** Indicators were developed from a literature review (including grey literature). A panel of 11-experts of a multidisciplinary background subjected the indicators to a two round consensus process. However, it was unclear if this occurred face-to face or remotely. |
| 68 | Is the medication being prescribed by generic name? | Development and validation of a new Prescription Quality Index (Hassan et al 2009) | **Literature based; Expert panel; Delphi Method:** Initial items were developed from an extensive review of the literature and discussion with experts. The first version of the PQI was piloted, and modified. Validity was examined using the Delphi Method in two separate surveys. |
| 67 | Is this drug the cheapest compared with other alternatives for the same indication? | Development and validation of a new Prescription Quality Index (Hassan et al 2009) | **Literature based; Expert panel; Delphi Method:** Initial items were developed from an extensive review of the literature and discussion with experts. The first version of the PQI was piloted, and modified. Validity was examined using the Delphi Method in two separate surveys. |
| 26 | Is this drug the least expensive alternative compared to others of equal utility? | A method for assessing drug therapy appropriateness [MAI]. (Hanlon et al 1992) | **Literature based; Expert panel:** These indicators were developed through a review of literature related to drug-related problems. A Clinical pharmacist and an Internist-geriatrician identified key elements based on this literature and experience and created the MAI criteria. |
| 2 | Patient and/or caregiver self-report of problems with cost | A Delphi process to address medication  appropriateness for older persons with  multiple chronic conditions (Fried et al 2016) | **Delphi method; literature based; research team:** These indicators were developed using a modified Delphi technique. Included ‘time-zero’ meeting (from RAND/UCLA appropriateness method) and core research team generated a candidate set of items for the panel to consider based on the results of the time zero meeting and literature review. The nine panellists represented the disciplines of nursing, medicine and pharmacy. |
| **Directions** | | | |
| **Indicator Number** | **Indicator** | **Source** | **Method of development** |
| 20 | Are the directions correct? | A method for assessing drug therapy appropriateness [MAI]. (Hanlon et al 1992) | **Literature based; Expert panel:** These indicators were developed through a review of literature related to drug-related problems. A Clinical pharmacist and an Internist-geriatrician identified key elements based on this literature and experience and created the MAI criteria. |
| 60 | Are the directions for administration correct? | Development and validation of a new Prescription Quality Index (Hassan et al 2009) | **Literature based; Expert panel; Delphi Method:** Initial items were developed from an extensive review of the literature and discussion with experts. The first version of the PQI was piloted, and modified. Validity was examined using the Delphi Method in two separate surveys. |
| 61 | Are the directions for administration practical? | Development and validation of a new Prescription Quality Index (Hassan et al 2009) | **Literature based; Expert panel; Delphi Method:** Initial items were developed from an extensive review of the literature and discussion with experts. The first version of the PQI was piloted, and modified. Validity was examined using the Delphi Method in two separate surveys. |
| 21 | Are the directions practical? | A method for assessing drug therapy appropriateness [MAI]. (Hanlon et al 1992) | **Literature based; Expert panel:** These indicators were developed through a review of literature related to drug-related problems. A Clinical pharmacist and an Internist-geriatrician identified key elements based on this literature and experience and created the MAI criteria. |
| 227 | Are the instructions given to the patient correct? | Selection of tools for reconciliation, compliance and appropriateness of treatment in patients with multiple chronic conditions (Lara et al 2012) | **Literature based; Expert panel:** Indicators were developed from a literature review (including grey literature). A panel of 11-experts of a multidisciplinary background subjected the indicators to a two round consensus process. However, it was unclear if this occurred face-to face or remotely. |
| 105 | Prescriber-patient: The prescriber gives information on the drug(s) to the patient—for example, on indications and directions for use | Prevalence of potentially inappropriate long term prescribing in general practice in the United  Kingdom, 1980–95: systematic literature review (Buetow et al 1996) | **Expert panel:** A panel of 10-experts used professional opinion to suggest consensus-based criteria. The discussions were recorded and transcribed. These were used by the research team to develop 19 implicit indicators covering five dimensions: indication, choice of drug, drug administration, communication, and review. |
| 107 | Prescriber-pharmacist: The prescription contains all the information needed for dispensing by a pharmacist | Prevalence of potentially inappropriate long term prescribing in general practice in the United  Kingdom, 1980–95: systematic literature review (Buetow et al 1996) | **Expert panel:** A panel of 10-experts used professional opinion to suggest consensus-based criteria. The discussions were recorded and transcribed. These were used by the research team to develop 19 implicit indicators covering five dimensions: indication, choice of drug, drug administration, communication, and review. |
| 314 | Is the patient/caregiver unclear about the medication regimen? | The Geriatric Medication Algorithm: A Pilot Study (Newton et al. 1994) | **Expert panel:** Two geriatric internists discussed medications of four elderly patients and a further internist recorded their implicit rules. An initial algorithm was developed and reviewed by a panel of five further internists. Final algorithm was tested in a resident outpatient clinic of a teaching hospital. |
| **Dosage/duration** | | | |
| **Indicator Number** | **Indicator** | **Source** | **Method of development** |
| 36 | Are dosage and dosage form adapted to the patient? | A method for assessing drug therapy appropriateness [MAI]. (Hanlon et al 1992) | **Literature based; Expert panel:** These indicators were developed through a review of literature related to drug-related problems. A Clinical pharmacist and an Internist-geriatrician identified key elements based on this literature and experience and created the MAI criteria. |
| 103 | Delivery: The formulation and route and method of delivery are designed to maximise compliance for an individual patient | Prevalence of potentially inappropriate long term prescribing in general practice in the United  Kingdom, 1980–95: systematic literature review (Buetow et al 1996) | **Expert panel:** A panel of 10-experts used professional opinion to suggest consensus-based criteria. The discussions were recorded and transcribed. These were used by the research team to develop 19 implicit indicators covering five dimensions: indication, choice of drug, drug administration, communication, and review. |
| 318 | Appropriateness of therapy: Dosage form/route | Measuring the Impact of Patient Counseling in the Outpatient Pharmacy Setting: Development and Implementation of the Counseling Models for the Kaiser Permanente AJSC Patient Consultation Study (Johnson et al 1995) | **Literature based ; Expert panel:** A multidisciplinary team developed screening criteria for determining high-risk patients. The team reviewed the literature and internal data concerning drug-related problems associated with a high likelihood of hospital admission. From this information, three screening criteria were developed. |
| 101 | Dose: The total daily amount of the drug prescribed falls within the range stated in the British National Formulary or the prescriber records the reason(s) why | Prevalence of potentially inappropriate long term prescribing in general practice in the United  Kingdom, 1980–95: systematic literature review (Buetow et al 1996) | **Expert panel:** A panel of 10-experts used professional opinion to suggest consensus-based criteria. The discussions were recorded and transcribed. These were used by the research team to develop 19 implicit indicators covering five dimensions: indication, choice of drug, drug administration, communication, and review. |
| 102 | Dosing frequency and duration: The dosing frequency and duration of the drug treatment fall within the ranges recommended in the British National Formulary or the prescriber records the reason(s) why | Prevalence of potentially inappropriate long term prescribing in general practice in the United  Kingdom, 1980–95: systematic literature review (Buetow et al 1996) | **Expert panel:** A panel of 10-experts used professional opinion to suggest consensus-based criteria. The discussions were recorded and transcribed. These were used by the research team to develop 19 implicit indicators covering five dimensions: indication, choice of drug, drug administration, communication, and review. |
| 317 | Appropriateness of Therapy: Drug regimen/dosage/strength | Measuring the Impact of Patient Counseling in the Outpatient Pharmacy Setting: Development and Implementation of the Counseling Models for the Kaiser Permanente AJSC Patient Consultation Study (Johnson et al 1995) | **Literature based ; Expert panel:** A multidisciplinary team developed screening criteria for determining high-risk patients. The team reviewed the literature and internal data concerning drug-related problems associated with a high likelihood of hospital admission. From this information, three screening criteria were developed. |
| 51 | If the dosage frequency is outside the range stated in the BNF, the prescriber gives a reason for prescribing it in the discharge summary. | Development and face validity of explicit indicators of appropriateness of long  term prescribing (Tully et al 2005) | **Literature based, research team, Nominal Group Technique:** Version 1 of the indicators was developed from both the earlier work of the authors and previously published indicators. Domains of appropriate prescribing had been developed by the authors, using the nominal group technique. Indicators were then refined for the hospital setting in the UK, operationalised using patient records by a member of the research team and a clinical pharmacist, refined further, operationalised with a further sample of patient records and refined again, before being subjected to face validity testing. |
| 77 | If the dosing frequency is outside the ranges stated in the BNF, the prescriber gives a valid reason | Indicators of the appropriateness of long term  prescribing in general practice in the United  Kingdom: consensus development, face and  content validity, feasibility, and reliability (Cantrill et al 1998) | **Nominal Group Technique:** 9-Panel member comprising multi-disciplinary background. Each indicator was discussed, and the list refined. A Delphi technique (two rounds) was used to assess face validity and content validity and to develop consensus. A minimum sample of 100 respondents per discipline was required for the survey. |
| 46 | If the duration of a drug is outside the range stated in the BNF, the prescriber gives a reason in the discharge summary. | Development and face validity of explicit indicators of appropriateness of long  term prescribing (Tully et al 2005) | **Literature based, research team, Nominal Group Technique:** Version 1 of the indicators was developed from both the earlier work of the authors and previously published indicators. Domains of appropriate prescribing had been developed by the authors, using the nominal group technique. Indicators were then refined for the hospital setting in the UK, operationalised using patient records by a member of the research team and a clinical pharmacist, refined further, operationalised with a further sample of patient records and refined again, before being subjected to face validity testing. |
| 50 | If the total daily dose is outside the range stated in the BNF, the prescriber gives a reason for prescribing it in the discharge summary. | Development and face validity of explicit indicators of appropriateness of long  term prescribing (Tully et al 2005) | **Literature based, research team, Nominal Group Technique:** Version 1 of the indicators was developed from both the earlier work of the authors and previously published indicators. Domains of appropriate prescribing had been developed by the authors, using the nominal group technique. Indicators were then refined for the hospital setting in the UK, operationalised using patient records by a member of the research team and a clinical pharmacist, refined further, operationalised with a further sample of patient records and refined again, before being subjected to face validity testing. |
| 76 | If the total daily dose is outside the range stated in the BNF, the prescriber gives a valid reason | Indicators of the appropriateness of long term  prescribing in general practice in the United  Kingdom: consensus development, face and  content validity, feasibility, and reliability (Cantrill et al 1998) | **Nominal Group Technique:** 9-Panel member comprising multi-disciplinary background. Each indicator was discussed, and the list refined. A Delphi technique (two rounds) was used to assess face validity and content validity and to develop consensus. A minimum sample of 100 respondents per discipline was required for the survey. |
| 19 | Is the dosage correct? | A method for assessing drug therapy appropriateness [MAI]. (Hanlon et al 1992) | **Literature based; Expert panel:** These indicators were developed through a review of literature related to drug-related problems. A Clinical pharmacist and an Internist-geriatrician identified key elements based on this literature and experience and created the MAI criteria. |
| 329 | Is the drug as currently given likely to be subtherapeutic or toxic, based on the dose, route and dosing interval for the age and renal status of the patient? | Reducing Polypharmacy in Extended care (Hamdy et al. 1995) | **Literature based:** These indicators were developed using guidelines from drug-regimen review criteria, used by consultant pharmacists. No other detail is provided and these were not subject to a consensus process. |
| 226 | Is the regimen schedule correct for this drug and patient? | Selection of tools for reconciliation, compliance and appropriateness of treatment in patients with multiple chronic conditions (Lara et al 2012) | **Literature based; Expert panel:** Indicators were developed from a literature review (including grey literature). A panel of 11-experts of a multidisciplinary background subjected the indicators to a two round consensus process. However, it was unclear if this occurred face-to face or remotely. |
| 232 | Is this the best administration route for the drug for this patient? | Selection of tools for reconciliation, compliance and appropriateness of treatment in patients with multiple chronic conditions (Lara et al 2012) | **Literature based; Expert panel:** Indicators were developed from a literature review (including grey literature). A panel of 11-experts of a multidisciplinary background subjected the indicators to a two round consensus process. However, it was unclear if this occurred face-to face or remotely. |
| 342 | Medication doses reflect age/renal status of older adult. | Evidence-Based Guideline  Improving Medication Management  for Older Adult Clients (Bergman-Evans, 2006) | **Literature based:** Not stated how these indicators were developed: paper is a review/evidence synthesis of other published indicators. Indicators not stated to have been subject to any consensus development. |
| 311 | Should the dosage be decreased? | The Geriatric Medication Algorithm: A Pilot Study (Newton et al. 1994) | **Expert panel:** Two geriatric internists discussed medications of four elderly patients and a further internist recorded their implicit rules. An initial algorithm was developed and reviewed by a panel of five further internists. Final algorithm was tested in a resident outpatient clinic of a teaching hospital. |
| 88 | Should the Dose Dose Frequency and/or Form of the Drug be Adjusted? | Prescribing Optimization Method for Improving Prescribing in Elderly Patients Receiving Polypharmacy Results of Application to Case Histories by General Practitioners (Drenth-van Maanen et al 2009) | **Research team; Literature based:** These indicators (originally listed in the form of questions with short evidence overview) were developed by the research team; the evidence overview is stated to be based on ‘available literature.’ |
| 302 | The patient is taking/receiving too much drug. | Pharmacist's Management of Drug-Related Problems: A Tool for Teaching and Providing Pharmaceutical Care (Winslade et al 1997) | **Expert panel**: The development of these indicators was initially based on two existing pharmaceutical care tools, which were modified significantly through practice models and experience. |
| 301 | The patient is taking/receiving too little drug. | Pharmacist's Management of Drug-Related Problems: A Tool for Teaching and Providing Pharmaceutical Care (Winslade et al 1997) | **Expert panel**: The development of these indicators was initially based on two existing pharmaceutical care tools, which were modified significantly through practice models and experience. |
| 319 | Appropriateness of Therapy: Quantity/duration | Measuring the Impact of Patient Counseling in the Outpatient Pharmacy Setting: Development and Implementation of the Counseling Models for the Kaiser Permanente AJSC Patient Consultation Study (Johnson et al 1995) | **Literature based; Expert panel:** A multidisciplinary team developed screening criteria for determining high-risk patients. The team reviewed the literature and internal data concerning drug-related problems associated with a high likelihood of hospital admission. From this information, three screening criteria were developed. |
| 297 | Any drug prescribed beyond the recommended duration, where treatment duration is well defined. | STOPP/START criteria for potentially inappropriate prescribing in older people:  version 2 (Mahony et al 2014) | **Literature based; Delphi Technique:**  *Phase 1*: Call for review of STOPP/START version 1 criteria and proposal of a new evidence-based criteria/removal of obsolete criteria.  *Phase 2*: Draft 1 of STOPP/START version 2 criteria.  *Phase 3*: Search of PubMed, Embase and Cochrane databases for systematic reviews, reviews and other references to support STOPP/START version 2 criteria.  *Phase 4*: Draft 2 of STOPP/START version 2 criteria, with support literature.  *Phase 5*: Delphi validation Round 1 (19 experts).  *Phase 6*: Draft 3 of STOPP/START version 2 criteria.  *Phase 7*: Delphi validation Round 2 (19 experts).  *Phase 8*: Draft 4 of STOPP/START version 2 criteria (final draft). |
| 78 | If the duration of treatment is outside the ranges stated in the BNF, the prescriber gives a valid reason | Indicators of the appropriateness of long term  prescribing in general practice in the United  Kingdom: consensus development, face and  content validity, feasibility, and reliability (Cantrill et al 1998) | **Nominal Group Technique:** 9-Panel member comprising multi-disciplinary background. Each indicator was discussed, and the list refined. A Delphi technique (two rounds) was used to assess face validity and content validity and to develop consensus. A minimum sample of 100 respondents per discipline was required for the survey. |
| 25 | Is the duration of therapy acceptable? | A method for assessing drug therapy appropriateness [MAI]. (Hanlon et al 1992) | **Literature based; Expert panel:** These indicators were developed through a review of literature related to drug-related problems. A Clinical pharmacist and an Internist-geriatrician identified key elements based on this literature and experience and created the MAI criteria. |
| **Duplication** | | | |
| **Indicator Number** | **Indicator** | **Source** | **Method of development** |
| 293 | >1 drug from same drug class | Prescribing Indicators for Evaluating Drug Use in  Nursing Homes (van Dijk et al 2003) | **Research team:** No mention of indicators being based on literature or subjected to expert consensus. |
| 327 | Are there duplications in the patient's drug therapy? That is, is the patient receiving two or more drugs from the same class (eg, verapamil and diltiazam)? Can the regimen be simplified? | Reducing Polypharmacy in Extended care (Hamdy et al. 1995) | **Literature based:** These indicators were developed using guidelines from drug-regimen review criteria, used by consultant pharmacists. No other detail is provided and these were not subject to a consensus process. |
| 230 | Is there a lack of therapeutic duplication? | Selection of tools for reconciliation, compliance and appropriateness of treatment in patients with multiple chronic conditions (Lara et al 2012) | **Literature based; Expert panel:** Indicators were developed from a literature review (including grey literature). A panel of 11-experts of a multidisciplinary background subjected the indicators to a two round consensus process. However, it was unclear if this occurred face-to face or remotely. |
| 35 | Is there any duplication? | A method for assessing drug therapy appropriateness [MAI]. (Hanlon et al 1992) | **Literature based; Expert panel:** These indicators were developed through a review of literature related to drug-related problems. A Clinical pharmacist and an Internist-geriatrician identified key elements based on this literature and experience and created the MAI criteria. |
| 65 | Is there unnecessary duplication with other drug(s)? | Development and validation of a new Prescription Quality Index (Hassan et al 2009) | **Literature based; Expert panel; Delphi Method:** Initial items were developed from an extensive review of the literature and discussion with experts. The first version of the PQI was piloted, and modified. Validity was examined using the Delphi Method in two separate surveys. |
| 340 | No duplications of medications are noted. | Evidence-Based Guideline  Improving Medication Management  for Older Adult Clients (Bergman-Evans, 2006) | **Literature based:** Not stated how these indicators were developed: paper is a review/evidence synthesis of other published indicators. Indicators not stated to have been subject to any consensus development. |
| 97 | Unnecessary duplications: Drugs from the same chemical or pharmacological class are not prescribed simultaneously | Prevalence of potentially inappropriate long term prescribing in general practice in the United  Kingdom, 1980–95: systematic literature review (Buetow et al 1996) | **Expert panel:** A panel of 10-experts used professional opinion to suggest consensus-based criteria. The discussions were recorded and transcribed. These were used by the research team to develop 19 implicit indicators covering five dimensions: indication, choice of drug, drug administration, communication, and review. |
| **General indicators** | | | |
| **Indicator Number** | **Indicator** | **Source** | **Method of development** |
| 288 | Adherence for all chronic medication was not checked or discussed during the past year (refill rate). Adherence for all new medication was not checked or discussed at first refill during the past year? | Older patients’ prescriptions screening in the community pharmacy: development of the Ghent Older People’s Prescriptions community Pharmacy Screening (GheOP3S) tool (Tommelein et al 2015) | **Literature based; RAND/UCLA appropriateness method:** It included (i) a round zero meeting (research team), (ii) a literature review, (iii) a first written Delphi round (multi-disciplinary 11-panelist group participated), (iv) a second face-to-face Delphi round based on the first round evaluation (only 8 panellists attended) and (v) finally, a selection of those items considered applicable in the contemporary community pharmacy practice (a panel of seven Belgian community pharmacists were invited to select those items that were applicable in the contemporary community pharmacy practice). |
| 315 | Does the patient need a compliance aid? | The Geriatric Medication Algorithm: A Pilot Study (Newton et al. 1994) | **Expert panel:** Two geriatric internists discussed medications of four elderly patients and a further internist recorded their implicit rules. An initial algorithm was developed and reviewed by a panel of five further internists. Final algorithm was tested in a resident outpatient clinic of a teaching hospital. |
| 199 | Patient has had no significant change in medications in the previous 90 days | Inappropriate Medication Use and Prescribing Indicators in Elderly Australians Development of a Prescribing Indicators Tool (Basger et al 2008) | **Literature based; Research team:** Common medical conditions for elderly Australians visiting the GP were identified; cross checked against drug treatments for these and then sub-optimal use of these drugs were identified. Data sources were based on expert review of evidence, approved product information, reference text books, latest international literature, Australian Consensus documents, and clinical practice guidelines from which 46 indicators were derived. |
| 233 | Medication Regimen Complexity Index | Development and psychometric evaluation of the  German version of the Medication Regimen  Complexity Index (MRCI-D) (Stange et al 2010) | **Unknown; Expert panel:** The English version of the MCRI was translated to German by two German pharmacists fluent in English. The two versions were compared and discrepancies discussed, with a medical psychologist, and a German version generated. This version was then back-translated by English pharmacists, discrepancies discussed and minor modifications made in accordance with the opinions of an expert in psychometric assessment and the author of the original MRCI. It was not detailed how the original English MCRI was developed. |
| 79 | When considering the patient’s total regimen, the dosing schedule is as simple as possible | Indicators of the appropriateness of long term  prescribing in general practice in the United  Kingdom: consensus development, face and  content validity, feasibility, and reliability (Cantrill et al 1998) | **Nominal Group Technique:** 9-Panel member comprising multi-disciplinary background. Each indicator was discussed, and the list refined. A Delphi technique (two rounds) was used to assess face validity and content validity and to develop consensus. A minimum sample of 100 respondents per discipline was required for the survey. |
| 90 | No failure to prescribe: Drugs are always prescribed when and how they should be, according to the recommendations in the British National Formulary | Prevalence of potentially inappropriate long term prescribing in general practice in the United  Kingdom, 1980–95: systematic literature review (Buetow et al 1996) | **Expert panel:** A panel of 10-experts used professional opinion to suggest consensus-based criteria. The discussions were recorded and transcribed. These were used by the research team to develop 19 implicit indicators covering five dimensions: indication, choice of drug, drug administration, communication, and review. |
| 289 | Polypharmacy patients (chronically taking five or more drugs) were not questioned about whether a clear medication scheme was available to him/her. | Older patients’ prescriptions screening in the community pharmacy: development of the Ghent Older People’s Prescriptions community Pharmacy Screening (GheOP3S) tool (Tommelein et al 2015) | **Literature based; RAND/UCLA appropriateness method:** It included (i) a round zero meeting (research team), (ii) a literature review, (iii) a first written Delphi round (multi-disciplinary 11-panelist group participated), (iv) a second face-to-face Delphi round based on the first round evaluation (only 8 panellists attended) and (v) finally, a selection of those items considered applicable in the contemporary community pharmacy practice (a panel of seven Belgian community pharmacists were invited to select those items that were applicable in the contemporary community pharmacy practice). |
| 341 | Client counseled on safety of nonprescription products. | Evidence-Based Guideline  Improving Medication Management  for Older Adult Clients (Bergman-Evans, 2006) | **Literature based:** Not stated how these indicators were developed: paper is a review/evidence synthesis of other published indicators. Indicators not stated to have been subject to any consensus development. |
| 338 | Goal of five medications or fewer is met. | Evidence-Based Guideline  Improving Medication Management  for Older Adult Clients (Bergman-Evans, 2006) | **Literature based:** Not stated how these indicators were developed: paper is a review/evidence synthesis of other published indicators. Indicators not stated to have been subject to any consensus development. |
| 343 | Patient is able to safely self-administer medications or a measure is in place to compensate for identified deficits. | Evidence-Based Guideline  Improving Medication Management  for Older Adult Clients (Bergman-Evans, 2006) | **Literature based:** Not stated how these indicators were developed: paper is a review/evidence synthesis of other published indicators. Indicators not stated to have been subject to any consensus development. |
| 286 | Availability of assistance in medication/health issues (by nurse, neighbour, children, etc.) was not checked nor discussed in frail older patients or older patients with reduced cognition, especially when taking drugs needing strict intake scheme. | Older patients’ prescriptions screening in the community pharmacy: development of the Ghent Older People’s Prescriptions community Pharmacy Screening (GheOP3S) tool (Tommelein et al 2015) | **Literature based; RAND/UCLA appropriateness method:** It included (i) a round zero meeting (research team), (ii) a literature review, (iii) a first written Delphi round (multi-disciplinary 11-panelist group participated), (iv) a second face-to-face Delphi round based on the first round evaluation (only 8 panellists attended) and (v) finally, a selection of those items considered applicable in the contemporary community pharmacy practice (a panel of seven Belgian community pharmacists were invited to select those items that were applicable in the contemporary community pharmacy practice). |
| 41 | Does the medication policy fits into the global care plan of this patient? | A method for assessing drug therapy appropriateness [MAI]. (Hanlon et al 1992) | **Literature based; Expert panel:** These indicators were developed through a review of literature related to drug-related problems. A Clinical pharmacist and an Internist-geriatrician identified key elements based on this literature and experience and created the MAI criteria. |
| 287 | The patient was not asked which aspects of pharmaceutical care could be improved for him/her (translated into practical questions for the specific patient: e.g. correct inhaler use and splitting tablets) | Older patients’ prescriptions screening in the community pharmacy: development of the Ghent Older People’s Prescriptions community Pharmacy Screening (GheOP3S) tool (Tommelein et al 2015) | **Literature based; RAND/UCLA appropriateness method:** It included (i) a round zero meeting (research team), (ii) a literature review, (iii) a first written Delphi round (multi-disciplinary 11-panelist group participated), (iv) a second face-to-face Delphi round based on the first round evaluation (only 8 panellists attended) and (v) finally, a selection of those items considered applicable in the contemporary community pharmacy practice (a panel of seven Belgian community pharmacists were invited to select those items that were applicable in the contemporary community pharmacy practice). |
| 85 | Which Drug(s) can be Withdrawn or Which Drug(s) is/are Inappropriate for this Patient? | Prescribing Optimization Method for Improving Prescribing in Elderly Patients Receiving Polypharmacy Results of Application to Case Histories by General Practitioners (Drenth-van Maanen et al 2009) | **Research team; Literature based:** These indicators (originally listed in the form of questions with short evidence overview) were developed by the research team; the evidence overview is stated to be based on ‘available literature.’ |
| 284 | Dispensation of over-the-counter medication (NSAID, ASA ...) was not added in the electronic patient record. | Older patients’ prescriptions screening in the community pharmacy: development of the Ghent Older People’s Prescriptions community Pharmacy Screening (GheOP3S) tool (Tommelein et al 2015) | **Literature based; RAND/UCLA appropriateness method:** It included (i) a round zero meeting (research team), (ii) a literature review, (iii) a first written Delphi round (multi-disciplinary 11-panelist group participated), (iv) a second face-to-face Delphi round based on the first round evaluation (only 8 panellists attended) and (v) finally, a selection of those items considered applicable in the contemporary community pharmacy practice (a panel of seven Belgian community pharmacists were invited to select those items that were applicable in the contemporary community pharmacy practice). |
| 335 | Medication list is complete and updated. | Evidence-Based Guideline  Improving Medication Management  for Older Adult Clients (Bergman-Evans, 2006) | **Literature based:** Not stated how these indicators were developed: paper is a review/evidence synthesis of other published indicators. Indicators not stated to have been subject to any consensus development. |
| 106 | Prescriber-prescriber: The medical record contains a comprehensive and accessible list of all prescribed drugs and regular over the counter drugs used by the patient | Prevalence of potentially inappropriate long term prescribing in general practice in the United  Kingdom, 1980–95: systematic literature review (Buetow et al 1996) | **Expert panel:** A panel of 10-experts used professional opinion to suggest consensus-based criteria. The discussions were recorded and transcribed. These were used by the research team to develop 19 implicit indicators covering five dimensions: indication, choice of drug, drug administration, communication, and review. |
| **Other inappropriate prescribing** | | | |
| **Indicator Number** | **Indicator** | **Source** | **Method of development** |
| 47 | If a drug is listed in the BNF as ‘less suitable for prescribing’, the prescriber gives a reason for prescribing it in the discharge summary | Development and face validity of explicit indicators of appropriateness of long  term prescribing (Tully et al 2005) | **Literature based, research team, Nominal Group Technique:** Version 1 of the indicators was developed from both the earlier work of the authors and previously published indicators. Domains of appropriate prescribing had been developed by the authors, using the nominal group technique. Indicators were then refined for the hospital setting in the UK, operationalised using patient records by a member of the research team and a clinical pharmacist, refined further, operationalised with a further sample of patient records and refined again, before being subjected to face validity testing. |
| 300 | The patient is taking/receiving the wrong drug or drug product. | Pharmacist's Management of Drug-Related Problems: A Tool for Teaching and Providing Pharmaceutical Care (Winslade et al 1997) | **Expert panel**: The development of these indicators was initially based on two existing pharmaceutical care tools, which were modified significantly through practice models and experience. |
| **Indication** | | | |
| **Indicator Number** | **Indicator** | **Source** | **Method of development** |
| 296 | Any drug prescribed without an evidence-based clinical indication. | STOPP/START criteria for potentially inappropriate prescribing in older people:  version 2 (Mahony et al 2014) | **Literature based; Delphi Technique:**  *Phase 1*: Call for review of STOPP/START version 1 criteria and proposal of a new evidence-based criteria/removal of obsolete criteria.  *Phase 2*: Draft 1 of STOPP/START version 2 criteria.  *Phase 3*: Search of PubMed, Embase and Cochrane databases for systematic reviews, reviews and other references to support STOPP/START version 2 criteria.  *Phase 4*: Draft 2 of STOPP/START version 2 criteria, with support literature.  *Phase 5*: Delphi validation Round 1 (19 experts).  *Phase 6*: Draft 3 of STOPP/START version 2 criteria.  *Phase 7*: Delphi validation Round 2 (19 experts).  *Phase 8*: Draft 4 of STOPP/START version 2 criteria (final draft). |
| 316 | Appropriateness of Therapy: Drug/indication | Measuring the Impact of Patient Counseling in the Outpatient Pharmacy Setting: Development and Implementation of the Counseling Models for the Kaiser Permanente AJSC Patient Consultation Study (Johnson et al 1995) | **Literature based ; Expert panel:** A multidisciplinary team developed screening criteria for determining high-risk patients. The team reviewed the literature and internal data concerning drug-related problems associated with a high likelihood of hospital admission. From this information, three screening criteria were developed. |
| 89 | Explicit and valid indication: The stated reason for prescribing the drug is upheld by the British National Formulary | Prevalence of potentially inappropriate long term prescribing in general practice in the United  Kingdom, 1980–95: systematic literature review (Buetow et al 1996) | **Expert panel:** A panel of 10-experts used professional opinion to suggest consensus-based criteria. The discussions were recorded and transcribed. These were used by the research team to develop 19 implicit indicators covering five dimensions: indication, choice of drug, drug administration, communication, and review. |
| 326 | Is the indication for which the drug was originally prescribed still present? | Reducing Polypharmacy in Extended care (Hamdy et al. 1995) | **Literature based:** These indicators were developed using guidelines from drug-regimen review criteria, used by consultant pharmacists. No other detail is provided and these were not subject to a consensus process. |
| 306 | Is there a specific indication for this drug? | The Geriatric Medication Algorithm: A Pilot Study (Newton et al. 1994) | **Expert panel:** Two geriatric internists discussed medications of four elderly patients and a further internist recorded their implicit rules. An initial algorithm was developed and reviewed by a panel of five further internists. Final algorithm was tested in a resident outpatient clinic of a teaching hospital. |
| 221 | Is there an explicit indication for the drug? | Selection of tools for reconciliation, compliance and appropriateness of treatment in patients with multiple chronic conditions (Lara et al 2012) | **Literature based; Expert panel:** Indicators were developed from a literature review (including grey literature). A panel of 11-experts of a multidisciplinary background subjected the indicators to a two round consensus process. However, it was unclear if this occurred face-to face or remotely. |
| 27 | Is there an indication | Appropriate prescribing for older people: a new tool for the general practitioner (Lenaerts et al 2013). | **Unknown:** There is no detail in the original source of how these indicators were developed. |
| 17 | Is there an indication for the drug? | A method for assessing drug therapy appropriateness [MAI]. (Hanlon et al 1992) | **Literature based; Expert panel:** These indicators were developed through a review of literature related to drug-related problems. A Clinical pharmacist and an Internist-geriatrician identified key elements based on this literature and experience and created the MAI criteria. |
| 6 | Medications for which there are no indication, including medications started at an earlier time for self-limited problems | A Delphi process to address medication  appropriateness for older persons with  multiple chronic conditions (Fried et al 2016) | **Delphi method; literature based; research team:** These indicators were developed using a modified Delphi technique. Included ‘time-zero’ meeting (from RAND/UCLA appropriateness method) and core research team generated a candidate set of items for the panel to consider based on the results of the time zero meeting and literature review. The nine panellists represented the disciplines of nursing, medicine and pharmacy. |
| 339 | Medications prescribed match established diagnosis. | Evidence-Based Guideline  Improving Medication Management  for Older Adult Clients (Bergman-Evans, 2006) | **Literature based:** Not stated how these indicators were developed: paper is a review/evidence synthesis of other published indicators. Indicators not stated to have been subject to any consensus development. |
| 44 | The indication for the drug in the discharge summary is upheld in the British National Formulary (BNF). | Development and face validity of explicit indicators of appropriateness of long  term prescribing (Tully et al 2005) | **Literature based, research team, Nominal Group Technique:** Version 1 of the indicators was developed from both the earlier work of the authors and previously published indicators. Domains of appropriate prescribing had been developed by the authors, using the nominal group technique. Indicators were then refined for the hospital setting in the UK, operationalised using patient records by a member of the research team and a clinical pharmacist, refined further, operationalised with a further sample of patient records and refined again, before being subjected to face validity testing. |
| 43 | The indication for the drug is recorded in the discharge summary. | Development and face validity of explicit indicators of appropriateness of long  term prescribing (Tully et al 2005) | **Literature based, research team, Nominal Group Technique:** Version 1 of the indicators was developed from both the earlier work of the authors and previously published indicators. Domains of appropriate prescribing had been developed by the authors, using the nominal group technique. Indicators were then refined for the hospital setting in the UK, operationalised using patient records by a member of the research team and a clinical pharmacist, refined further, operationalised with a further sample of patient records and refined again, before being subjected to face validity testing. |
| 42 | The indication for the drug is recorded in the inpatient medical record. | Development and face validity of explicit indicators of appropriateness of long  term prescribing (Tully et al 2005) | **Literature based, research team, Nominal Group Technique:** Version 1 of the indicators was developed from both the earlier work of the authors and previously published indicators. Domains of appropriate prescribing had been developed by the authors, using the nominal group technique. Indicators were then refined for the hospital setting in the UK, operationalised using patient records by a member of the research team and a clinical pharmacist, refined further, operationalised with a further sample of patient records and refined again, before being subjected to face validity testing. |
| 70 | The indication for the drug is recorded in the medical record and upheld by the BNF | Indicators of the appropriateness of long term  prescribing in general practice in the United  Kingdom: consensus development, face and  content validity, feasibility, and reliability (Cantrill et al 1998) | **Nominal Group Technique:** 9-Panel member comprising multi-disciplinary background. Each indicator was discussed, and the list refined. A Delphi technique (two rounds) was used to assess face validity and content validity and to develop consensus. A minimum sample of 100 respondents per discipline was required for the survey. |
| 298 | The patient is taking/receiving a drug for which there is no valid indication. | Pharmacist's Management of Drug-Related Problems: A Tool for Teaching and Providing Pharmaceutical Care (Winslade et al 1997) | **Expert panel**: The development of these indicators was initially based on two existing pharmaceutical care tools, which were modified significantly through practice models and experience. |
| **Drug-drug interactions** | | | |
| **Indicator Number** | **Indicator** | **Source** | **Method of development** |
| 22 | Are there clinically significant drug-drug interactions? | A method for assessing drug therapy appropriateness [MAI]. (Hanlon et al 1992) | **Literature based; Expert panel:** These indicators were developed through a review of literature related to drug-related problems. A Clinical pharmacist and an Internist-geriatrician identified key elements based on this literature and experience and created the MAI criteria. |
| 312 | Are there drug interactions or additive side effects? | The Geriatric Medication Algorithm: A Pilot Study (Newton et al. 1994) | **Expert panel:** Two geriatric internists discussed medications of four elderly patients and a further internist recorded their implicit rules. An initial algorithm was developed and reviewed by a panel of five further internists. Final algorithm was tested in a resident outpatient clinic of a teaching hospital. |
| 32 | Are there significant clinical interactions | A method for assessing drug therapy appropriateness [MAI]. (Hanlon et al 1992) | **Literature based; Expert panel:** These indicators were developed through a review of literature related to drug-related problems. A Clinical pharmacist and an Internist-geriatrician identified key elements based on this literature and experience and created the MAI criteria. |
| 96 | Co-prescribing: Drugs are prescribed simultaneously to make use of beneficial interactions between them | Prevalence of potentially inappropriate long term prescribing in general practice in the United  Kingdom, 1980–95: systematic literature review (Buetow et al 1996) | **Expert panel:** A panel of 10-experts used professional opinion to suggest consensus-based criteria. The discussions were recorded and transcribed. These were used by the research team to develop 19 implicit indicators covering five dimensions: indication, choice of drug, drug administration, communication, and review. |
| 323 | Interaction: Drug-drug | Measuring the Impact of Patient Counseling in the Outpatient Pharmacy Setting: Development and Implementation of the Counseling Models for the Kaiser Permanente AJSC Patient Consultation Study (Johnson et al 1995) | **Literature based ; Expert panel:** A multidisciplinary team developed screening criteria for determining high-risk patients. The team reviewed the literature and internal data concerning drug-related problems associated with a high likelihood of hospital admission. From this information, three screening criteria were developed. |
| 54 | If a potentially hazardous drug-drug combination is prescribed, the prescriber gives details of the interaction and recommended action in both the inpatient record and the discharge summary | Development and face validity of explicit indicators of appropriateness of long  term prescribing (Tully et al 2005) | **Literature based, research team, Nominal Group Technique:** Version 1 of the indicators was developed from both the earlier work of the authors and previously published indicators. Domains of appropriate prescribing had been developed by the authors, using the nominal group technique. Indicators were then refined for the hospital setting in the UK, operationalised using patient records by a member of the research team and a clinical pharmacist, refined further, operationalised with a further sample of patient records and refined again, before being subjected to face validity testing. |
| 74 | If a potentially hazardous drug-drug combination is used, the prescriber shows knowledge of the hazard | Indicators of the appropriateness of long term  prescribing in general practice in the United  Kingdom: consensus development, face and  content validity, feasibility, and reliability (Cantrill et al 1998) | **Nominal Group Technique:** 9-Panel member comprising multi-disciplinary background. Each indicator was discussed, and the list refined. A Delphi technique (two rounds) was used to assess face validity and content validity and to develop consensus. A minimum sample of 100 respondents per discipline was required for the survey. |
| 33 | Are there significant clinical interactions between medications | A method for assessing drug therapy appropriateness [MAI]. (Hanlon et al 1992) | **Literature based; Expert panel:** These indicators were developed through a review of literature related to drug-related problems. A Clinical pharmacist and an Internist-geriatrician identified key elements based on this literature and experience and created the MAI criteria. |
| 229 | Is there a lack of relevant interactions between this and other drugs? | Selection of tools for reconciliation, compliance and appropriateness of treatment in patients with multiple chronic conditions (Lara et al 2012) | **Literature based; Expert panel:** Indicators were developed from a literature review (including grey literature). A panel of 11-experts of a multidisciplinary background subjected the indicators to a two round consensus process. However, it was unclear if this occurred face-to face or remotely. |
| 151 | Patient has no clinically significant medication interactions (agreement between two medication interaction databases)* | Validation of prescribing appropriateness criteria for older Australians using the RAND/UCLA  appropriateness method (Basger et al 2012) | **Literature based; RAND/UCLA Appropriateness method:** Commonly used medications and conditions were reviewed from 2008 paper in 2011, and new/changed evidence was noted for evaluation by the expert panel. Round 1 consensus consisted of 15 multidisciplinary panel members for initial rating. Round 2 (face to face meeting) consisted on 12 panel members. |
| 198 | Patient has no significant medication interactions (agreement between two medication interaction databases) | Inappropriate Medication Use and Prescribing Indicators in Elderly Australians Development of a Prescribing Indicators Tool (Basger et al 2008) | **Literature based; Research team:** Common medical conditions for elderly Australians visiting the GP were identified; cross checked against drug treatments for these and then sub-optimal use of these drugs were identified. Data sources were based on expert review of evidence, approved product information, reference text books, latest international literature, Australian Consensus documents, and clinical practice guidelines from which 46 indicators were derived. |
| 95 | Potentially serious interactions: Potentially hazardous drug-drug interactions, as recorded in the British National Formulary, are avoided or the prescriber explicitly states that the potential benefits outweigh the risks | Prevalence of potentially inappropriate long term prescribing in general practice in the United  Kingdom, 1980–95: systematic literature review (Buetow et al 1996) | **Expert panel:** A panel of 10-experts used professional opinion to suggest consensus-based criteria. The discussions were recorded and transcribed. These were used by the research team to develop 19 implicit indicators covering five dimensions: indication, choice of drug, drug administration, communication, and review. |
| 305 | The patient is experiencing a drug-drug, drug-food, or drug-laboratory interaction. | Pharmacist's Management of Drug-Related Problems: A Tool for Teaching and Providing Pharmaceutical Care (Winslade et al 1997) | **Expert panel**: The development of these indicators was initially based on two existing pharmaceutical care tools, which were modified significantly through practice models and experience. |
| **Medication review** | | | |
| **Indicator Number** | **Indicator** | **Source** | **Method of development** |
| 110 | Frequency of review: The drug treatment is reviewed by the general practitioner at least once a year or in accordance with the guidelines in the British National Formulary | Prevalence of potentially inappropriate long term prescribing in general practice in the United  Kingdom, 1980–95: systematic literature review (Buetow et al 1996) | **Expert panel:** A panel of 10-experts used professional opinion to suggest consensus-based criteria. The discussions were recorded and transcribed. These were used by the research team to develop 19 implicit indicators covering five dimensions: indication, choice of drug, drug administration, communication, and review. |
| 82 | Patient’s medication has been reviewed within the previous 12 months | Indicators of the appropriateness of long term  prescribing in general practice in the United  Kingdom: consensus development, face and  content validity, feasibility, and reliability (Cantrill et al 1998) | **Nominal Group Technique:** 9-Panel member comprising multi-disciplinary background. Each indicator was discussed, and the list refined. A Delphi technique (two rounds) was used to assess face validity and content validity and to develop consensus. A minimum sample of 100 respondents per discipline was required for the survey. |
| **Specific safety issues** | | | |
| **Indicator Number** | **Indicator** | **Source** | **Method of development** |
| 276 | 1. Use of two or more agents with anticholinergic activity OR use of a highly anticholinergic agent | Development of evidence-based Australian medication-related indicators of potentially preventable hospitalisations: a modified RAND  appropriateness method (Caughey et al 2014) | **Literature based; RAND/UCLA Appropriateness Method:** A modified RAND appropriateness method was used for the development of medication-related indicators of potentially preventable hospitalisations, which has characteristics of both the Delphi and Nominal Group Techniques. In addition,  indicators were developed for gastrointestinal disorders (associated with high prevalence and morbidity in Australia). A literature review, assessment of the strength of the supporting evidence base, an initial face and content validity assessment by an expert panel (convenience sample of 8 pharmacists), followed by an independent assessment of indicators by an expert clinical panel across various disciplines (352 clinical experts), using an online survey took place. |
| 246 | 1. Patient aged ≥65 years 2. Use of two or more agents with anticholinergic activity OR use of an agent with high anticholinergic activity | Development of evidence-based Australian medication-related indicators of potentially preventable hospitalisations: a modified RAND  appropriateness method (Caughey et al 2014) | **Literature based; RAND/UCLA Appropriateness Method:** A modified RAND appropriateness method was used for the development of medication-related indicators of potentially preventable hospitalisations, which has characteristics of both the Delphi and Nominal Group Techniques. In addition,  indicators were developed for gastrointestinal disorders (associated with high prevalence and morbidity in Australia). A literature review, assessment of the strength of the supporting evidence base, an initial face and content validity assessment by an expert panel (convenience sample of 8 pharmacists), followed by an independent assessment of indicators by an expert clinical panel across various disciplines (352 clinical experts), using an online survey took place. |
| 257 | 1. Use of two or more agents with low-to-moderate anticholinergic activity; OR use of a highly anticholinergic agent | Development of evidence-based Australian medication-related indicators of potentially preventable hospitalisations: a modified RAND  appropriateness method (Caughey et al 2014) | **Literature based; RAND/UCLA Appropriateness Method:** A modified RAND appropriateness method was used for the development of medication-related indicators of potentially preventable hospitalisations, which has characteristics of both the Delphi and Nominal Group Techniques. In addition,  indicators were developed for gastrointestinal disorders (associated with high prevalence and morbidity in Australia). A literature review, assessment of the strength of the supporting evidence base, an initial face and content validity assessment by an expert panel (convenience sample of 8 pharmacists), followed by an independent assessment of indicators by an expert clinical panel across various disciplines (352 clinical experts), using an online survey took place. |
| 183 | Patient is not taking more than one medication with anticholinergic activity (q) | Inappropriate Medication Use and Prescribing Indicators in Elderly Australians Development of a Prescribing Indicators Tool (Basger et al 2008) | **Literature based; Research team:** Common medical conditions for elderly Australians visiting the GP were identified; cross checked against drug treatments for these and then sub-optimal use of these drugs were identified. Data sources were based on expert review of evidence, approved product information, reference text books, latest international literature, Australian Consensus documents, and clinical practice guidelines from which 46 indicators were derived. |
| 204 | High sodium-containing medications: effervescent tablets and powders – Panadol® Soluble, Berocca®, Supradyn®, Aspro Clear®, Ural®, Alka-Seltzer®, Eno®, vitamin C (sodium ascorbate), Gaviscon®, Mylanta® | Inappropriate Medication Use and Prescribing Indicators in Elderly Australians Development of a Prescribing Indicators Tool (Basger et al 2008) | **Literature based; Research team:** Common medical conditions for elderly Australians visiting the GP were identified; cross checked against drug treatments for these and then sub-optimal use of these drugs were identified. Data sources were based on expert review of evidence, approved product information, reference text books, latest international literature, Australian Consensus documents, and clinical practice guidelines from which 46 indicators were derived. |
| 7 | Medications identified by expert opinion as being inappropriate for older persons, such as those included in the Beers and STOPP lists | A Delphi process to address medication  appropriateness for older persons with  multiple chronic conditions (Fried et al 2016) | **Delphi method; literature based; research team:** These indicators were developed using a modified Delphi technique. Included ‘time-zero’ meeting (from RAND/UCLA appropriateness method) and core research team generated a candidate set of items for the panel to consider based on the results of the time zero meeting and literature review. The nine panellists represented the disciplines of nursing, medicine and pharmacy. |
| 217 | Medications that may cause dyspepsia: aspirin, bisphosphonates, calcium channel antagonists, corticosteroids, dopaminergic agents, erythromycin, iron, nitrates, NSAIDs, potassium chloride, tetracycline[51,55,68] | Inappropriate Medication Use and Prescribing Indicators in Elderly Australians Development of a Prescribing Indicators Tool (Basger et al 2008) | **Literature based; Research team:** Common medical conditions for elderly Australians visiting the GP were identified; cross checked against drug treatments for these and then sub-optimal use of these drugs were identified. Data sources were based on expert review of evidence, approved product information, reference text books, latest international literature, Australian Consensus documents, and clinical practice guidelines from which 46 indicators were derived. |
| 331 | Review of medications reveals no conflict with Beers’ list (Beers et al., 1991). | Evidence-Based Guideline  Improving Medication Management  for Older Adult Clients (Bergman-Evans, 2006) | **Literature based:** Not stated how these indicators were developed: paper is a review/evidence synthesis of other published indicators. Indicators not stated to have been subject to any consensus development. |
| **Under-prescribing** | | | |
| **Indicator Number** | **Indicator** | **Source** | **Method of development** |
| 83 | Is the Patient Undertreated and is Additional Medication Indicated? | Prescribing Optimization Method for Improving Prescribing in Elderly Patients Receiving Polypharmacy Results of Application to Case Histories by General Practitioners (Drenth-van Maanen et al 2009) | **Research team; Literature based:** These indicators (originally listed in the form of questions with short evidence overview) were developed by the research team; the evidence overview is stated to be based on ‘available literature.’ |
| 223 | Is there a lack of diagnoses or symptoms recorded in the medical history that do not have drug treatments but could have it? | Selection of tools for reconciliation, compliance and appropriateness of treatment in patients with multiple chronic conditions (Lara et al 2012) | **Literature based; Expert panel:** Indicators were developed from a literature review (including grey literature). A panel of 11-experts of a multidisciplinary background subjected the indicators to a two round consensus process. However, it was unclear if this occurred face-to face or remotely. |
| 299 | The patient requires drug therapy for an indication and is not receiving/taking this therapy. | Pharmacist's Management of Drug-Related Problems: A Tool for Teaching and Providing Pharmaceutical Care (Winslade et al 1997) | **Expert panel**: The development of these indicators was initially based on two existing pharmaceutical care tools, which were modified significantly through practice models and experience. |
